# Evaluating gene expression and biomarkers for mastitis resistance in Barki sheep

**DOI:** 10.1038/s41598-025-04515-1

**Published:** 2025-06-20

**Authors:** Ahmed Adel El-Sayed, Gehad Elshopakey, Ahmed Ateya

**Affiliations:** 1 Department of Animal Health and Poultry, Animal and Poultry Production Division, Desert Research Center (DRC), Cairo, 11753 Egypt; 2 Department of Clinical Pathology, Faculty of Veterinary Medicine, Mansoura University, Mansoura, 35516 Egypt; 3 Department of Development of Animal Wealth, Faculty of Veterinary Medicine, Mansoura University, Mansoura, 35516 Egypt

**Keywords:** Barki sheep, Antioxidants, Immunity, Single nucleotide polymorphisms, Gene expression, Mastitis, Biochemistry, Immunology, Molecular biology, Physiology, Biomarkers

## Abstract

The aim of this study was monitoring health status of mastitic Barki ewes using candidate gene approach, gene expression and serum profile of inflammatory and antioxidant markers. A total of 70 ewes were allocated into two equal-sized groups: healthy and ewes have a history of mastitis. DNA sequencing of *IFN-γ* (365-bp), *IL-4* (285-bp), *TNF-α* (273-bp), *MYD88* (660-bp), *CCL5* (360-bp), *TLR4* (256-bp), *TLR9* (414-bp), *LTF* (299-bp), *PRLR* (891-bp), *CAT* (300-bp), *GPX1* (221-bp), *Keap1* (360-bp), *OXSR1* (357-bp), *ATOX1* (433-bp), *GST* (480-bp) and *Nrf2* (340-bp) revealed single nucleotide polymorphisms (SNPs) between healthy and mastitic ewes. Levels of *IFN-γ*,* IL-4*,* TNF-α*,* MYD88*, *CCL5*,* TLR4*,* TLR9*,* LTF*,* PRLR*, *Keap1* and *OXSR1* genes expression were significantly up-regulated in ewes affected with mastitis than resistant ones. Meanwhile *CAT*, *GPX1*, *ATOX1*, *GST*, and *Nrf2* genes elicited an opposite trend. There is a significant elevation of activity of AST, LDH, Hp, CP, SAA, IgG, MDA, and NO levels (*P* < 0.05), along with reduction of total protein, albumin, GSH, GPx, catalase and SOD (*P* < 0.05) in mastitic ewes. The findings of this study supported the hypothesis that SNPs in immune and antioxidant genes could be important genetic markers for mastitis susceptibility or resistance in Barki ewes. The examined genes’ gene expression profiles may also be utilized as surrogate biomarkers to establish an efficient management regimen and forecast the period of time at which a disease is most likely to manifest.

**Supplementary Information:**

The online version contains supplementary material available at 10.1038/s41598-025-04515-1.

## Introduction

Egypt’s North-Western Coastal Zone depends heavily on Barki sheep, which are called after the Libyan area Barka and number 470,000. They live in severe desert areas, a lack of vegetation, and heat stress because they can adapt well to difficult circumstances^1^. Mastitis is one of the most significant issues affecting Barki ewes, and it is characterized by significant financial losses, including a decrease in milk production, a fall in the quality of the final product, and hygienic circumstances that make the final product unfit for human use^2^. Changes in the physical, chemical, pathological, and bacterial characteristics of milk and glandular tissues are characteristics of mastitis^3,4^. According to etiopathological findings and observations, the disease is frequently categorized as subclinical, acute, sub-acute, chronic, or gangrenous^5^. It is well recognized that inadequate management and cleanliness, teat injuries, and broken milking equipment speed up the entry of pathogenic microorganisms and the disease’s growth^6^.

Research on oxidative stress in ruminant medicine is still in its early stages. Although oxidative stress has been linked to several diseases, much remains unknown regarding its impact on ruminant production and health. Measuring the amount of antioxidants, vitamins, and enzymes in biological samples is another useful way to detect oxidative stress^7^. Antioxidants work together to counteract oxidative damage, so measuring one antioxidant does not provide a reliable picture of antioxidant capability^8^. As a result, numerous methods for estimating total antioxidant capacity have been devised. It has recently been found that monitoring the gene expression of antioxidant indicators is a useful way to monitor animal health, with the main goal being to predict a herd’s vulnerability to production illnesses^9^.

The liver is primarily responsible for producing the acute phase proteins (APPs), a class of blood proteins that prevent pathological damage, aid in homeostasis restoration, and, without the use of antibodies, restrict the growth of pathogens in infected animals^10^. APP levels are influenced by a wide range of pathogenic (like viral and non-infectious diseases) and physiological (like nutrition, age, sex, pregnancy, breastfeeding, and environmental factors) factors^11^. APPs’ association with the degree of sickness makes them potentially useful as prognostic indicators and flock health markers^12^.

Variations in the expression of several regulatory enzymes in intermediate metabolism can be used to improve genetic selection for cattle’s resilience to harsh conditions^13^. Metabolic regulation involves the transcriptional control of gene networks, which are groups of DNA segments that interact with nuclear receptors or transcription factors to regulate the levels of important enzymes in cells. These “global” interactions can influence how quickly the genes in the network are translated into mRNA. The broad discipline of genomics investigates putative genes at the level of mRNA, subnetworks, or the complete genome^14^.

To the best of our knowledge, little is known about the gene expression profiles, APPs alterations, proinflammatory cytokines, oxidative stress biomarkers, and SNPs associated with Barki sheep mastitis. This study’s goal was to look into possible genetic polymorphisms and differentially expressed genes in Barki sheep, as well as oxidative stress biomarkers, proinflammatory cytokines, and APPs changes linked to mastitis infection. Our study introduced new genetic markers and putative candidate genes for identifying mastitis infection in Barki sheep, suggesting that genetic variability between animals exists. These markers may be used as effective proxies for mastitis in Barki sheep and open promising opportunities to control the disease through selective breeding programs.

## Results

### Physical examination

Clinically, no mammary abnormalities were evident in any healthy Barki ewes with normal milk characteristics. Barki ewes with mastitis had elevated udder temperatures when compared to the body’s temperature, both evaluated by palpation. The mean rectal temperature of this group was 41.5 °C and expressed pain when the udders were manipulated. Milk was yellowish and dense. The majority of animals in this group also had edema and nodules in the udder and were apathetic.

### PCR-DNA sequencing of immunity and antioxidant genes

PCR-DNA sequencing of the following sequences: *IFN-γ* (365-bp), *IL-4* (285-bp), *TNF-α* (273-bp), *MYD88* (660-bp), *CCL5* (360-bp), *TLR4* (256-bp), *TLR9* (414-bp), *LTF* (299-bp), *PRLR* (891-bp), *CAT* (300-bp), *GPX1* (221-bp), *Keap1* (360-bp), *OXSR1* (347-bp), *ATOX1* (433-bp), *GST* (480-bp) and *Nrf2* (340-bp) revealed nucleotide sequence variations in the form of SNPs between mastitic and healthy Barki ewes. The nucleotide sequence variation of the genes under inquiry was compared with reference sequences available in GenBank, resistant sheep, and afflicted ewes to validate the eleven SNPs that were discovered (available in the GenBank with accession numbers gb|PP567235|, gb|PP567236|, gb|PP567237|, gb|PP567238|, gb|PP567239|, gb|PP567240|, gb|OR900038|, gb|OR900039|, gb|OR900040|, gb|PP575806|, gb|PP575807|, gb|PP575808|, gb|PP575809|, gb|PP575810|, gb|PP575811|, gb|PP575812|, gb|PP575813|, gb|PP575814|, gb|PP575815|, gb|PP575816|, gb|PP575817|, gb|PP575818|, gb|PP575819|, gb|PP596786|, gb|PP596787|, gb|PP596788|, gb|PP596789|, gb|PP596790|, gb|PP596791|, gb|PP596792|, gb|PP596793|, gb|PP596794|, gb|PP596795|).

All of the discovered SNPs were approved based on the reference gene sequences from GenBank and the DNA sequence differences between the immunological and antioxidant markers analysed in the sheep under study (Figures S1–S16). The immunological and antioxidant markers under study demonstrate the exonic region differences that led to coding mutations between the mastitic and healthy sheep in Table 1. Thirty seven of the forty-eight SNPs found in the immunological and antioxidant genes were non-synonymous, whereas the other eleven were synonymous.

**Table 1:** Distribution of snps, type of mutation in immune and antioxidant genes in healthy and mastitic ewes.

Gene	SNPs	Healthy	Reproductive disorders	Total	Chi square value X^2^	*P*-value	Chi square value X^2^	*P*-value	Type of mutation	Amino acid number and type
*IFN-γ*	C41T	18	–	18/70	4.94	< 0. 05	24.32	< 0.005	Non-synonymous	14 S to F
	T97C	–	31	31/70			55.64	< 0.005	Non-synonymous	33 L to F
	T193C	21	–	21/70			30.00	< 0.005	Non-synonymous	65 W to R
	A241G	–	28	28/70			46.67	< 0.005	Non-synonymous	81 S to G
*IL-4*	A41T	22	–	22/70	59.04	< 0.005	32.08	< 0.005	Non-synonymous	14 K to M
	A82G	29	–	29/70			49.51	< 0.005	Non-synonymous	28 I to V
*TNF-α*	T244C	–	19	19/70	19.90	< 0.005	26.08	< 0.005	Non-synonymous	82 W to R
*MYD88*	C53T	–	22	22/70	12.98	< 0.005	32.08	< 0.005	Non-synonymous	18 S to F
	A218G	–	33	33/70			62.43	< 0.005	Non-synonymous	73 K to R
	A256G	–	17	17/70			22.45	< 0.005	Non-synonymous	86 M to V
	A342G	29	–	29/70			49.51	< 0.005	Synonymous	114 Q
	C458A	–	26	26/70			41.36	< 0.005	Non-synonymous	153 P to H
	T461C	21	–	21/70			30.00	< 0.005	Non-synonymous	154 L to P
	T528C	26	–	26/70			41.36	< 0.005	Synonymous	176 H
	A570G	–	24	24/70			36.52	< 0.005	Synonymous	190 K
*CCL5*	C62T	–	22	22/70	51.26	< 0.005	32.08	< 0.005	Non-synonymous	25 F to L
	C144G	–	35	35/70			70.00	< 0.005	Non-synonymous	52 P to A
	T153C	–	21	21/70			30.00	< 0.005	Non-synonymous	55 F to L
	C185A	27	–	27/70			43.95	< 0.005	Non-synonymous	65 H to Q
	G294T	–	22	22/70			32.08	< 0.005	Non-synonymous	102 A to S
*TLR4*	A91G	–	21	21/70	49.94	< 0.005	30.00	< 0.005	Non-synonymous	31 K to E
	A134G	–	18	18/70			24.23	< 0.005	Non-synonymous	45 D to G
	G143C	24	–	24/70			36.52	< 0.005	Non-synonymous	48 S to T
	C178T	–	31	31/70			55.64	< 0.005	Synonymous	60 L
	T229G	–	24	24/70			36.52	< 0.005	Non-synonymous	77 Y to D
*TLR9*	C72T	20	–	20/70	32.39	< 0.005	38.89	< 0.005	Synonymous	24 S
	G89A	23	–	23/70			34.26	< 0.005	Non-synonymous	30 R to Q
	T133G	18	–	18/70			24.23	< 0.005	Non-synonymous	45 S to A
	G201A	–	28	28/70			46.67	< 0.005	Synonymous	67 T
	T321C	22	–	22/70			32.08	< 0.005	Synonymous	107 L
*LTF*	A73G	–	13	13/70	37.69	< 0.005	15.96	< 0.005	Intronic	–
	G104A	26	–	26/70			41.36	< 0.005		
	A142T	34	–	34/70			66.11	< 0.005		
	C148T	–	19	19/70			26.08	< 0.005		
	G156A	26	–	26/70			41.36	< 0.005		
	C162T	33	–	33/70			62.43	< 0.005		
	C187T	–	22	22/70			30.00	< 0.005		
	G232A	–	24	24/70			36.52	< 0.005		
	T246C	31	–	31/70			55.64	< 0.005		
	G252T	15	–	15/70			19.09	< 0.005		
*PRLR*	G219A	–	24	24/70	26.57	< 0.005	36.52	< 0.005	Synonymous	73 S
	A276G	23	–	23/70			34.26	< 0.005	Synonymous	92 P
	T459C	–	26	26/70			41.36	< 0.005	Synonymous	153 D
	C585G	–	18	18/70			46.82	< 0.005	Non-synonymous	195 S to R
*CAT*	T23 C	–	22	22/70	6.18	< 0. 05	32.08	< 0.005	Non-synonymous	8 I to T
	C71A	–	28	28/70			46.67	< 0.005	Non-synonymous	24 A to E
	C206T	30	–	30/70			52.50	< 0.005	Non-synonymous	69 T to M
*GPX1*	C43G	23	–	23/70	7.83	< 0.05	34.26	< 0.005	Non-synonymous	15 R to G
	C85T	–	26	26/70			41.36	< 0.005	Non-synonymous	29 R to C
	G178C	–	18	18/70			24.23	< 0.005	Non-synonymous	60 E to Q
*KEAP1*	C113G	–	15	15/70	29.98	< 0.005	19.09	< 0.005	Non-synonymous	38 P to R
	A254G	24	–	24/70			36.52	< 0.005	Non-synonymous	85 Y to C
	C275T	31	–	31/70			55.64	< 0.005	Non-synonymous	92 P to L
*OXSR1*	C220 T	28	–	28/70	31.71	< 0.005	57.39	< 0.005	Non-synonymous	74 L to F
*ATOX1*	G373C	34	–	34/70	41.49	< 0.005	66.11	< 0.005	Non-synonymous	125 V to L
*GST*	C30T	21	–	21/70	8.92	< 0.005	24.23	< 0.005	Synonymous	10 N
	T56G T57G	–	28 15	28/70 15/70			46.67 19.09	< 0.005	Non-synonymous	19 L to R
*Nrf2*	A191G	–	28	28/70	31.71	< 0.005	46.67	< 0.005	Non-synonymous	64 H to R

*IFN-γ* interferon gamma, *IL-4* iterleukin-4, *TNF-α* tumor necrosis factor-alpha, *MYD88* myeloid differentiation primary response 88, *CCL5* chemokine (C-C motif) ligand 5, *TLR4* toll-like receptor 4, *TLR9* toll-like receptor 9, *LTF* lactoferrin, *PRLR* prolactin receptor, *CAT* catalase, *GPX1* glutathione peroxidase 1, *KEAP1* Kelch-like ECH-associated protein 1, *OXSR1* oxidative stress responsive kinase 1, *ATOX1* antioxidant 1 copper chaperone 1, *GST* glutathione S transferase, *Nrf2* nuclear factor-erythroid factor 2-related factor.

A = Alanine; C = Cisteine; D = Aspartic acid; E = Glutamic acid; F = Phenylalanine; G = Glycine; H = Histidine; I = Isoleucine; K = Lysine; L = Leucine; M = Methionine; N = Asparagine; P = Proline; Q = Glutamine; R = Argnine; S = Serine; T = Threonine; V = Valine; W = Tryptophan and Y = Tyrosine.

A significant difference was detected in the frequencies of all examined genes SNPs among mastitic and healthy ewes (*p* < 0.005). Chi-square analysis was carried out for comparison of the distribution of all identified SNPs in all genes between mastitic and healthy ewes. Total chi-square value showed significant variation among the identified SNPs in all genes between resistant and affected animals (*p* < 0.05) (Table 1).

Results of Linear Discriminant Model Analysis yielded a significant Wilks’ Lambda value of 0. 02 at *P* < 0.005 indicating that the gene-level averages provided significant discrimination between the two health groups. Table below showed the specific gene contribution the most to the discriminant function and it showed that genes contributed the most to the discriminant function; *TNF-α* and *CAT* are associated mainly with health ewes, while *LTF*,* IL-4* and *MYD88* are associated mainly with mastitic ewes.

Results of Linear Discriminant Model Analysis yielded a significant Wilks’ Lambda value of 0. 02 at *P* < 0.005 indicating that the gene-level averages provided significant discrimination between the two studied groups. Table 2 showed the specific gene contribution the most to the discriminant function and it showed that genes contributed the most to the discriminant function; *TNF-α* and *CAT* are associated mainly with health ewes, while *LTF*,* IL-4* and *MYD88* are associated mainly with mastitic ewes.

**Table 2:** The standardized canonical discriminant function coefficients of each gene used in DA.

Gene	Standardized canonical discriminant function coefficients
*IFN-γ*	−0 0.120
*IL-4*	0.283
*TNF-α*	− 0.538
*MYD88*	0.251
*CCL5*	− 0.060
*TLR4*	− 0.148
*TLR9*	0.043
*LTF*	0.329
*PRLR*	0.037
*CAT*	− 0.509
*GPX1*	− 0.095
*KEAP1*	0.037
*OXSR1*	0.288
*ATOX1*	0.732
*GST*	− 0.064
*Nrf2*	− 0.179

-IFN-γ**–** Interferon gamma; IL-4– Iterleukin-4; TNF- α **–** Tumor necrosis factor- alpha; MYD88– Myeloid differentiation primary response 88; CCL5– Chemokine (C-C motif) ligand 5; TLR4– Toll-like receptor 4; TLR9– Toll-like receptor 9; LTF**–** Lactoferrin; PRLR**–** Prolactin receptor; CAT**–** Catalase; GPX1 = Glutathione peroxidase 1; KEAP1 = Kelch-like ECH-associated protein 1; OXSR1 = Oxidative Stress Responsive Kinase 1; ATOX1 = antioxidant 1 copper chaperone 1; GST**–** Glutathione S transferase; and Nrf2– Nuclear factor-erythroid factor 2-related factor.

Table 3 showed the discriminant analysis for classification of type of genes and healthy status. The classification results showed that the model correctly classified 100% of the cases overall either of healthy ewes or mastitic ewes. These results indicate that the SNP markers included in the model possess a good level of discriminatory power and may be useful as potential genetic indicators for mastitis susceptibility in sheep.

**Table 3:** Discriminant analysis for classification of type of genes and healthy status of examined ewes.

		Predicted group membership		Total
		Healthy	Diseases	
Count	Healthy	35	0	35
	Diseased	0	35	35
%	Healthy	100.0	0.0	100.0
	Diseased	0.0	100.0	100.0

### Gene expression pattern of immune and antioxidant markers

**Fig. 1:**
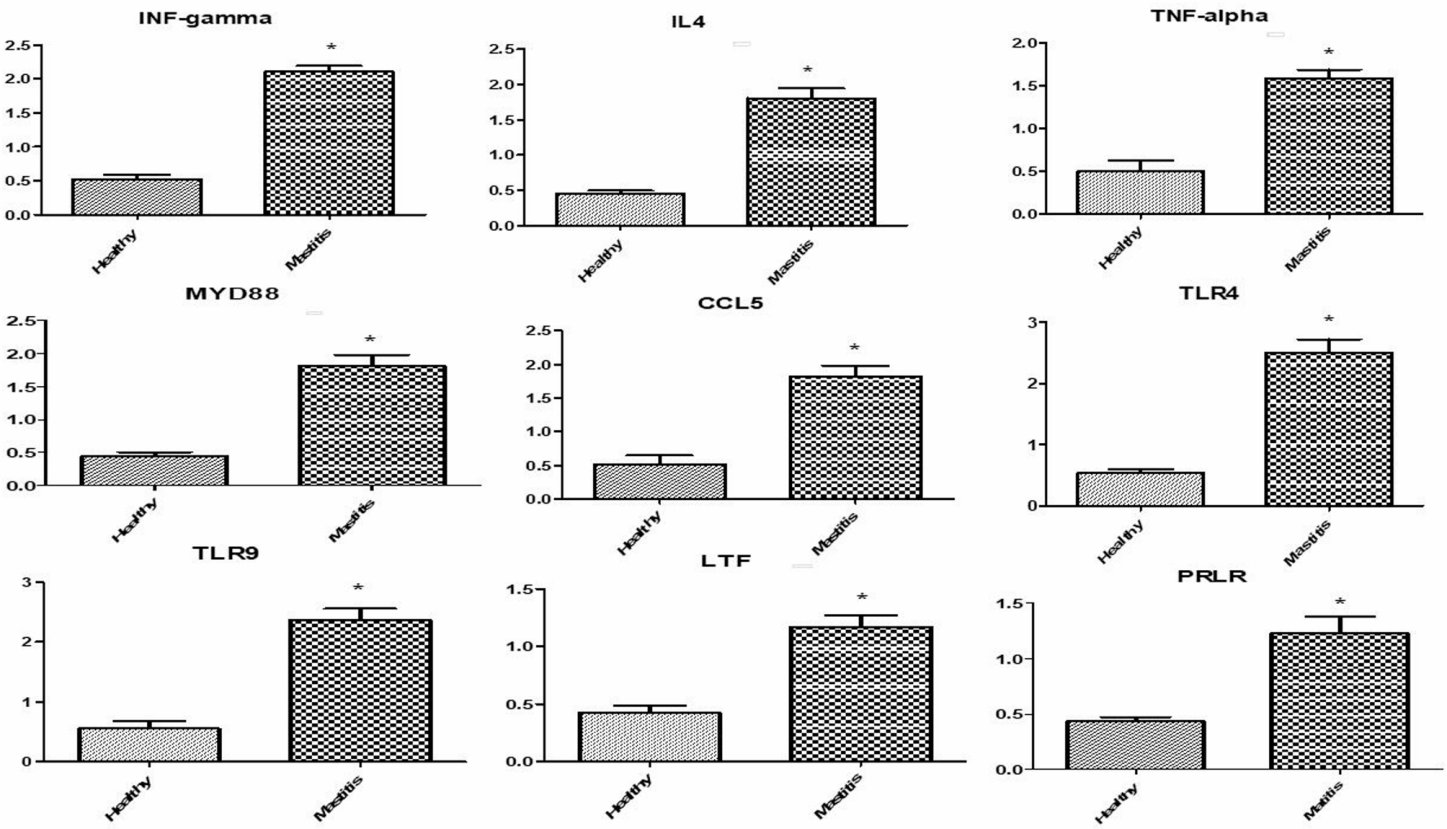
Relative expression patterns of immunity genes in the healthy and mastitis affected Barki ewes. Results are expressed as means ± SEM. *P < 0.05. *IFN-γ* interferon gamma, *IL-4* iterleukin-4, *TNF-α* tumor necrosis factor-alpha, *MYD88* myeloid differentiation primary response 88, *CCL5* chemokine (C-C motif) ligand 5, *TLR4* toll-like receptor 4, *TLR9* toll-like receptor 9, *LTF* lactoferrin, *PRLR* prolactin receptor.

**Fig. 2:**
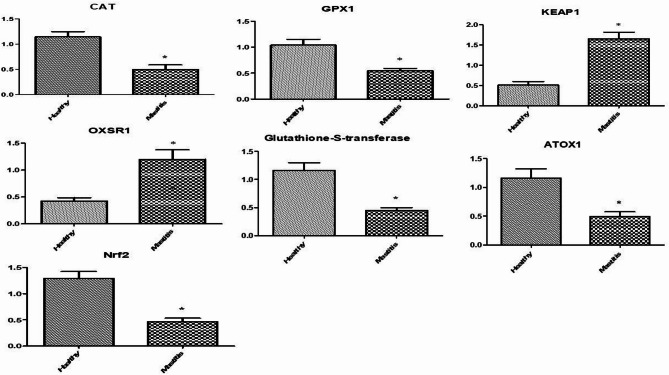
Relative expression patterns of antioxidant genes in the healthy and mastitis affected Barki ewes. Results are expressed as means ± SEM. *P < 0.05. *CAT* catalase, *GPX1* glutathione peroxidase 1, *Keap1* Kelch-like ECH-associated protein 1, *OXSR1* oxidative stress responsive kinase 1, *ATOX1* antioxidant 1 copper chaperone 1, *GST* glutathione S transferase, *Nrf2* nuclear factor-erythroid factor 2-related factor.

Figures 1 and 2 showed the immune and antioxidant marker gene expression profiles. Mastitis-affected ewes had considerably higher levels of *IFN-γ*,* IL-4*,* TNF-α*,* MYD88*, *CCL5*,* TLR4*,* TLR9*,* LTF*, and *PRLR* gene expression than resistant ones. Mastitis-affected sheep had significantly reduced expression levels of the genes *CAT*,* GPX1*,* ATOX1*,* GST*, and *Nrf2* compared to resistant ewes. However, there was a noticeable up-regulation of the *Keap1* and *OXSR1* genes in mastitic ewes. *Nrf2* is the most up-regulated gene (1.2967 ± 0.13) and *OXSR1* is the most down-regulated gene (0.4240 ± 0.06) in healthy Barki ewes. *TLR4* is the most up-regulated gene (2.5040 ± 0.21) in mastitis Barki ewes, whereas *GST* is the most down-regulated gene (0.4490 ± 0.05).

### Serum biochemical, inflammatory findings and oxidative stress/antioxidant markers

As illustrated in Table 4, the obtained data demonstrated significantly higher activities of AST and LDH (*P* < 0.001), with lower serum levels of total protein and albumin (*P* < 0.05, *P* < 0.01) in mastitic ewes than that of healthy controls. However, serum level of globulin (*P* < 0.001) was statistically elevated in mastitic ewes compared the control animals. No significantly valuable differences in serum ALT and ALP activates between the groups. To assess the mechanisms involved in the progression and damage of mammary gland tissue during mastitis, some serum inflammatory markers were estimated in our study (Table 5). The Hp, CP, SAA, and IgG levels (*P* < 0.001) were found to be highly elevated in the serum of mastitic ewes unlike that of the healthy controls. The mammary gland inflammation during the progression of mastitis caused the generation of an accentuated ROS and impairment of antioxidant molecules confirmed in our results by higher MDA, and NO levels (*P* < 0.001), along with reduction of GSH (*P* < 0.001), GPx (*P* < 0.001) catalase (*P* < 0.01), and SOD (*P* < 0.001) in mastitic ewes compared to the control non-infected one (Table 3).

**Table 4:** Serum biochemical parameters in healthy and mastitic ewes.

Parameters	Healthy ewes	Mastitic ewes	*P*-value
ALT (U/L)	18.42 ± 1.52	18.78 ± 2.13	NS
AST (U/L)	63.61 ± 4.90	92.89 ± 4.69	***
ALP (U/L)	169.14 ± 6.51	176.43 ± 9.35	NS
LDH (U/L)	234.67 ± 39.21	709.67 ± 50.06	***
T. protein (g/dL)	6.07 ± 0.51	7.71 ± 0.16	*
Albumin (g/dL)	3.50 ± 0.13	2.69 ± 0.11	**
Globulin (g/dL)	2.57 ± 0.44	5.02 ± 0.12	***

Data were represented as Mean ± SD. NS: Non-significant, *P < 0.05, **P < 0.01, ***P < 0.001.

*ALT* alanine aminotransferase, *AST* aspartate aminotransferase, *ALP* alkaline phosphatase, *LDH* lactate dehydrogenase.

**Table 5:** Serum inflammatory markers and oxidative stress/antioxidant parameters in healthy and mastitic ewes.

Parameters	Healthy ewes	Mastitic ewes	*P*-value
Haptoglobin (mg/dL)	28.66 ± 2.41	84.88 ± 6.09	***
Ceruloplasmin (mg/dL)	13.73 ± 1.79	50.49 ± 4.49	***
Amyloid A (ng/mL)	30.27 ± 1.55	72.61 ± 5.26	***
IgG (mg/dL)	16.24 ± 2.62	34.39 ± 2.56	***
MDA (nmol/L)	5.08 ± 1.05	17.75 ± 1.76	***
NO (µmol /L)	5.49 ± 0.39	11.04 ± 0.45	***
GSH (mg/dL)	6.55 ± 0.76	3.14 ± 0.51	***
GPx (mU/L)	36.59 ± 3.51	24.95 ± 2.98	***
Catalase (U/L)	438.5 ± 18.2	397.6 ± 10.6	**
SOD (U/L)	269.11 ± 10.63	147.39 ± 19.92	***

Data were represented as mean ± SD. **P < 0.01, ***P < 0.001.

*IgG* immunoglobulin G, *MDA* malondialdehyde, *NO* nitric oxide, *GSH* reduced glutathione, *GPx* glutathione peroxidase, *SOD* superoxide dismutase.

### Correlation between gene expression pattern and serum profile of immune and antioxidant markers

**Fig. 3:**
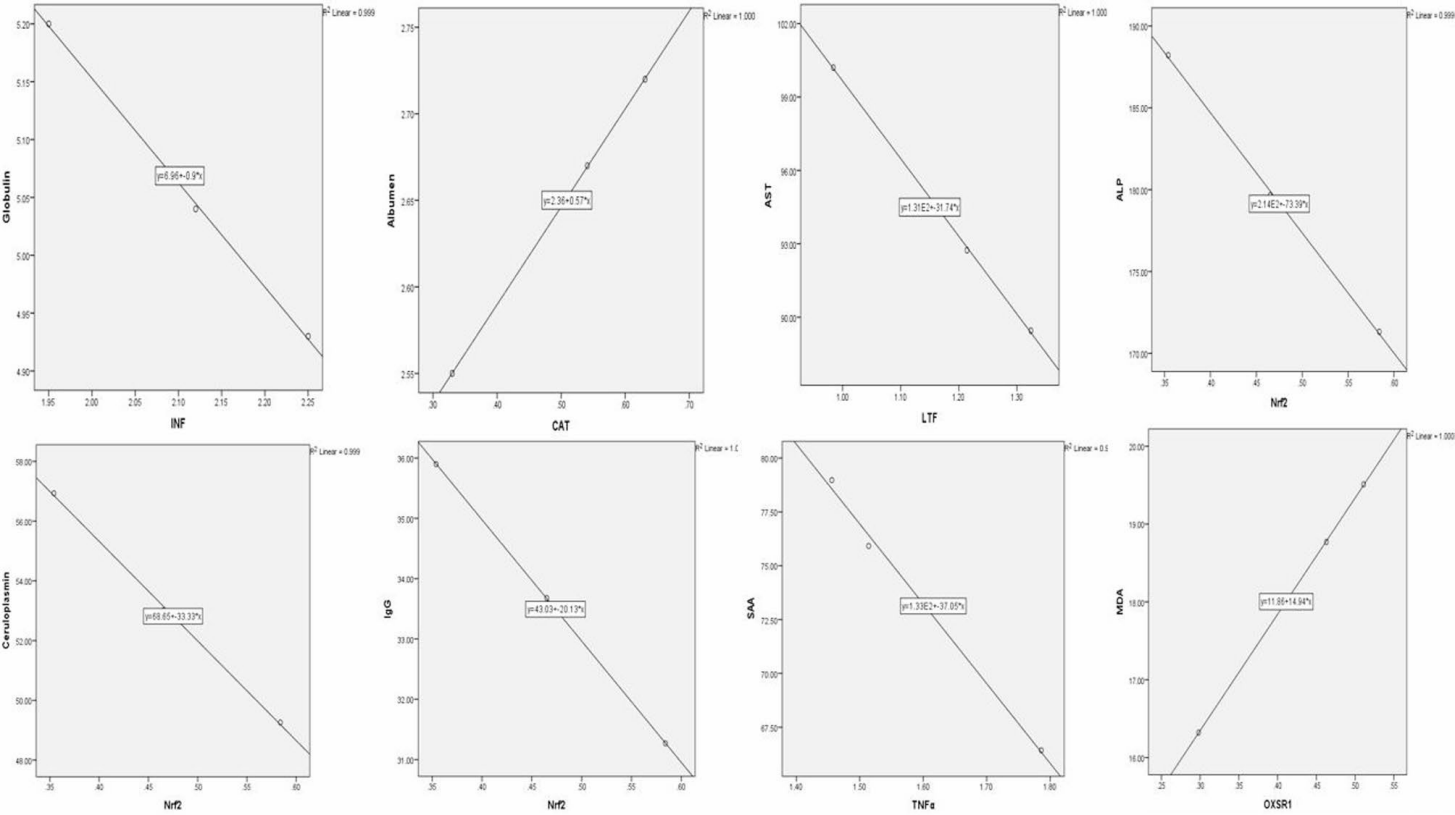
Correlation between mRNA levels and serum profile of immune and antioxidant markers in mastitis affected Barki ewes. *IFN-γ* interferon gamma, *TNF-α* tumor necrosis factor-alpha, *LTF* lactoferrin, *CAT* catalase, *OXSR1* oxidative stress responsive kinase 1, *Nrf2* nuclear factor-erythroid factor 2-related factor.

The serum levels of ALP, CP, IgG were negatively correlated with mRNA levels of *Nrf2* (*r*= -1 and *p* = 0.018, *r*= -0.99 and *p* = 0.02, *r*= -1and *p* = 0.002, respectively), serum levels of AST were negatively correlated with mRNA levels of *LTF* (*r*= -1 and *p* = 0.012), serum levels of globulin were negatively correlated with mRNA levels of *INF-γ* (*r*= -1 and *p* = 0.014), serum levels of SAA were negatively correlated with mRNA levels of *TNFα* (*r*= -0.998 and *p* = 0.04), serum levels of albumen were positively correlated with mRNA levels of *CAT* (*r* = 1 and *p* = 0.003) and serum levels of NO were negatively correlated with mRNA levels of *OXSR1* (*r*= -1 and *p* = 0.008) (Fig. 3).

## Discussion

To the best of our knowledge, no study has directly connected genetic variations, serum biochemical, antioxidant, and inflammatory markers (APPs) to the risk of mastitis in Barki sheep. Therefore, the aim of this study was to evaluate biochemical, antioxidant, and serum inflammatory indicators as well as genetic variations associated with Barki sheep’s mastitis susceptibility. It is yet unclear how inflammation during mastitis damages the tissue of the mammary glands. It is well known that free radicals such O_2_.-, H_2_O_2_, and OH contribute to inflammatory reactions that enhance vascular permeability and leukocyte movement^15^.

### Genetic polymorphisms of immune and antioxidant genes

PCR-DNA sequencing for the following genes: *IFN-γ* (365-bp), *IL-4* (285-bp), *TNF-α* (273-bp), *MYD88* (660-bp), *CCL5* (360-bp), *TLR4* (256-bp), *TLR9* (414-bp), *LTF* (299-bp), *PRLR* (891-bp), *CAT* (300-bp), *GPX1* (221-bp), *Keap1* (360-bp), *OXSR1* (347-bp), *ATOX1* (433-bp), *GST* (480-bp) and *Nrf2* (340-bp). To our knowledge, this is the first study that elicits nucleotide sequence variations in inflammatory and antioxidant genes to the possibility of mastitis resistance or susceptibility in Barki sheep. An intriguing discovery emerged from comparing our findings with a matching GenBank reference sequence: the polymorphisms discovered in the genes under study are presented here for the first time. However, Chu et al. (2007)^16^ demonstrated a substantial correlation between sheep high prolificacy and the *PRL* locus. Mastitis resistance and milk SCC in cows have been associated with the *LTF* gene^17^. Ateya et al. (2016)^18^ caused the *LTF* gene to be sequenced in both mastitis-affected and -healthy (resistant) pigs. The results demonstrated that PCR-DNA sequencing genetic screening identified twelve SNPs in the bovine *LTF* gene associated with mastitis susceptibility in Holstein dairy cows. The relationship between the LTF gene polymorphism in dairy goats and their physical traits and milk composition was described by Guo et al. (2010)^19^. There was a substantial correlation between *TLR4* variants and mastitis susceptibility in sheep^20^ and cattle^21,22^.

In another study for exploring gene polymorphisms and its relatedness with mastitis susceptibility, Elmaghraby et al. (2018)^23^ showed that there was no correlation between the TLR9 gene polymorphism and the likelihood of developing mastitis in Holstein cattle, and that there were no SNPs found separating the susceptible from the resistant animals. Wojdak-Maksymiec et al. (2013)^24^ outlined a connection between clinical mastitis in dairy cattle and variations in the *TNF-α* and *LTF* genes.

Mutation is the main mechanism of adaptation and selection^25^[52]. Mastitic ewes have different coding DNA sequences than healthy ewes because all of the immunological and antioxidant indicators under investigation in this case had exonic region alterations. In the antioxidant and immunological genes, there were 48 SNPs; 11 of them were synonymous, while 37 were non-synonymous. Natural selection usually targets animals with non-synonymous mutations because they change the sequence of proteins^25^. Non-synonymous SNPs cause genetic variation that modifies the encoded amino acid at the mutant position, which can change the mutated protein’s structure and function^26^. Selection on synonymous mutations was thought to be either nonexistent or very weak for a very long time^25^. Our study found polymorphisms based on translated DNA sequence to be more valuable in order to accurately characterise the examined immune and antioxidant genes at the molecular level and to comprehend the physiological differences in resistance/susceptibility between normal and mastitic ewes.

### Transcript levels of immune and antioxidant genes

In the current investigation, qRT-PCR was used to measure the levels of antioxidant (*CAT*,* GPX1*,* Keap1*,* OXSR1*,* ATOX1*,* GST*, and *Nrf2*) and immune (*IFN-γ*,* IL-4*,* TNF-α*,* MYD88*,* CCL5*,* TLR4*,* TLR9*,* LTF*, and *PRLR*) genes in Barki ewes that were resistant and non-resistant to mastitis. According to our research, immunological markers were more highly expressed in affected ewes than in resistant ewes; however, antioxidant genes exhibited the opposite tendency.

Prior research investigated gene polymorphism in domestic animals using genetic markers such as single nucleotide polymorphism (SNP) and restriction fragment length polymorphism (RFLP)^17^. On the other hand, our goal in using SNP genetic markers and gene expression to investigate gene polymorphism was to rectify the limitations of previous studies. Thus, in the mastitis-free and -affected ewes, the regulation mechanisms of *IFN-γ*,* IL-4*,* TNF-α*,* MYD88*,* CCL5*,* TLR4*,* TLR9*,* LTF*,* PRLR*,* CAT*,* GPX1*,* Keap1*,* OXSR1*,* ATOX1*,* GST*, and *Nrf2* are well known. To the best of our knowledge, there are remarkably few studies examining the gene expression profile of antioxidant and immunological markers associated with mastitis. Darwish et al.^27^ elicited that levels of *IL-5*,* IL-6*,* IL1-ß*,* TNF α*,* TLR4* and *Tollip (Toll interacting protein)* were shown to be substantially more up-regulated in postpartum disorder-affected sheep than in resistant ones, although the patterns of *SOD* and *CAT* genes showed the reverse tendency. Bonnefont et al. (2021)^28^ studied differential expression of mammary epithelial cells in response to *Staphylococcus aureus* mastitis in sheep. There was an increase in the RNA level of the chemokines *CCL20*,* CXCL3*,* TNF-α*, and *CXCL8* (*IL8*).

The up-regulation expression profile of the *LTF* gene for mastitis resistance in Damascus goats was found by Yakan et al. (2018)^29^ in terms of the gene expression profile of immunological and antioxidant indicators during mastitis in ruminants. Pisoni et al. (2010)^30^ found that goats with *Staphylococcus aureus* mastitis were significantly more likely than healthy goats to have elevated levels of pro-inflammatory cytokines and chemokines. The cytokine receptors (*IL-1a*,* lymphotoxin alpha*, *granulocyte chemotactic protein (CXCL6)*, and *IL-2 receptor gamma*) genes are also up-regulated in masitic does. In addition, Wu et al. (2015)^31^ found that, when comparing mastitis tissue in Holstein cattle to normal tissue, *TLR4*,* MyD88*,* IL-6*, and *IL-10* were up-regulated, whereas *CD14*,* TNF-α*,* MD-2*,* IL-β*,* NF-κB*, and *IL-12* were considerably down-regulated. Asadpour et al. (2021)^32^ investigated the differential expression of antioxidant genes during clinical mastitis in cows caused by Escherichia coli and S. aureus. It found that *SOD* expression was significantly up-regulated in mastitis milk caused by *S. aureus* as opposed to *E. coli*. Furthermore, compared to *S. aureus*, the mRNA levels of *GPx* in mastitis milk caused by E. coli were noticeably overexpressed. Fonseca et al. (2009)^33^ found that mastitis in dairy calves was linked to an increased expression profile of the genes for *IL-2*,* IL-4*,* IL-6*,* IL-8*,* IL-10*,* IFN-γ*, and *TNF-α*.

### Role of investigated genes in incidence of mastitis

Interferons are a multigene family of inducible cytokines. This group includes interferon gamma (IFN-γ), which is critical for the innate host response against intracellular pathogens like mycobacteria^32^. As part of a protective type 1-like T-cell response, it has been proposed that the release of IFN-γ after the pathogen’s first entry into the host plays a crucial role in controlling infection and disease manifestation^33,34^. Serum cytokines such IL-4, TNF-α, and IFN-γ also serve as indirect indications in inflammatory conditions^35^. Moreover, IL-4 stimulates the humoral immune response, or the formation of Th0 and Th2 cells^36^.

One of the primary pro-inflammatory cytokines in the immune response is TNF-α. TNF-α, in conjunction with other elements, promotes the growth, development, and function of several immune system cells, including B and T lymphocytes, natural killer (NK) lymphocytes, and lymphokine-activated killer (LAK) cells^34^. Furthermore, TNF-α induces the release of multiple additional cytokines [48]. The TNF-α gene has three introns and four exons, and it is located on chromosome BTA23q22 ^37^. Although expressed in many different types of mammalian cells, macrophages and monocytes express it most strongly. The bacterial cell wall’s lipopolysaccharide (LPS) induces these phagocytic cells to produce TNF-α. When LPS activates macrophages, there is a threefold increase in TNF-α gene expression, a 100-fold increase in mRNA levels, and a 10,000-fold increase in protein release^38^.

The prolactin receptor, encoded by the *PRLR* gene, is a member of the growth hormone/prolactin receptor gene family that shares some sequence similarities^39^. Prolactin and growth hormone receptors, as well as receptors for members of the cytokine superfamily, are homologous^40^. Previous studies have suggested that PRLR may be a gene related to a goat’s or sheep’s reproductive traits^41^. Glycoprotein lactoferrin (LTF) is an iron-binding protein that is structurally similar to iron-transport protein transferrin^42^. The antibacterial and anti-infective properties of LF have been demonstrated^43^. *LTF* gene expression in epithelial cells is 20 times higher than in leukocytes, indicating that *LTF* gene expression levels in ruminants may be a significant signal for mastitis resistance. This is because the majority of somatic cells in ruminants are formed of epithelial cells as a result of milk synthesis processes^44^.

The bulk of TLR molecules’ ectodomains have been found to have leucine-rich repeat domain (LRR) domains, which are essential elements that distinguish pattern-recognition receptors (PAMPs) at play^45^. Based on examinations of SNP distribution in *TLR* coding regions in many animals, non-synonymous SNPs are more prevalent in sequences encoding LRR domains^46^. The recognition ability of the molecule for exogenous pathogens may be substantially altered by non-synonymous SNPs in LRR domains^47^. CpG dinucleotides that are unmethylated in bacterial DNA are recognised by TLR9 ^48^. MYD88, a mediator of TLR9, appears to play a role in the genetic resistance of certain sheep diseases^35^. MYD88, a mediator of Toll-Like Receptor 9 (TLR9), appears to play a role in the genetic resistance of certain sheep diseases^35^. All TLRs, with the exception of TLR3, depend on Myeloid Differentiation Factor 88 (MyD88) to link TLR recognition of bacteria to NFkB activation and cytokine production^49^. The chemokine CCL5, which is regulated upon activation of normal T-cell expressed and secreted (RANTES) in response to inflammatory signals, is expressed by a variety of cells, including blood lymphocytes^50^. It regulates the trafficking and activation of both inflammatory and non-inflammatory cells^51,52^ and is connected to the acute phase response^51,52^.

Reactive oxygen species (ROS) can be scavenged or detoxified by antioxidants, which can also prevent ROS from being produced or sequester transition metals, which are the source of free radicals^53^. The enzymatic and non-enzymatic antioxidant defences that the body produces include catalase, glutathione S transferase (GST), and endogenous glutathione peroxidase (GPx)^54^. The copper metallochaperone protein ATOX1 is encoded by the *ATOX1* gene^55^. ATOX1 protected cells from reactive oxygen species. ATOX1 is necessary for maintaining copper homeostasis because it moves copper from the cytosol to the transporters ATP7A and ATP7B^56^.

OSR1 which is encoded by (*OXSR1*) gene, controls downstream kinases in response to environmental stress^57^. The lowest pattern was seen at calving in dromedary camels when comparing the OXSR1 expression profile during the peri-parturient phase to the times at calving and (+ 14)^58^. The primary inducible defence against oxidative stress is the Keap1-Nrf2 stress response system, which regulates the production of cytoprotective genes^59^. Keap1 serves as a substrate adapter for cullin-based E3 ubiquitin ligase, which normally inhibits the transcriptional activity of Nrf2 through ubiquitination and proteasomal degradation^60^. This could account for the *Keap1* and *Nrf2* genes’ opposing pattern of expression in our investigation.

The expression patterns of immunological and antioxidant markers are significantly altered in mastitis-affected ewes. This alteration might be related to the severe inflammation that kills the affected tissue and makes phagocytic cells produce cytotoxic radicals and pro-inflammatory cytokines^61,62^. Furthermore, the immune system is weakened by the overabundance of reactive oxygen species (ROS) when there is insufficient total antioxidant^63^. A large number of leukocytes travel from the circulation to the mammary gland infection site after bacterial infection, where they participate in the inflammatory process and the body’s response to the invading pathogens^64^. This activates the mammary gland’s immune system. This suggests that overexpressed genes associated to immunity are necessary for host defense.

### Serum markers

Compared to healthy ewes, mastitic ewes had significantly higher serum activity of AST and LDH. Our results are consistent with previous studies^36^but contrast with earlier research^65^which shown that there were no appreciable variations in AST activity between the healthy and mastitic groups. Stressful settings may have contributed to the significantly greater AST and LDH activities of mastitic Barki ewes when compared to healthy ones.

The blood levels of albumin and total protein in mastitic ewes were considerably lower than in non-mastitic ewes. These results corroborated those found in buffaloes^37^in cattle^65^ and in camels^36^. However, they differed from those found in ewes^38,39^ in Sahel goats^40^, in Indigeous cows^42^, in affected Crossbred cattle and^43^ in buffaloes. There was no discernible difference between mastitis and healthy ones, according to the authors of the later study. In contrast^44,45^, found that the serum total protein level was significantly higher in the mastitic group than in the healthy ones. The observed disparity could potentially be attributed to the reduced albumin levels subsequent to the immune reaction to the udder infection^66^. Moreover, it is believed that albumin, a negative APP that carries out several physiological tasks, including antioxidant activity, and raises vascular permeability to reach inflamed tissues, is a biomarker of immunological inflammation^66^.

Mastitic ewes had a considerably higher serum level of globulin than healthy ones. These results were similar to those reported in Red Sokoto goats^45^ and in camels^36^but they differed from those reported by utilizing^44^ who found no significant differences in globulin concentrations between healthy and mastitic Sahel goats and cows, respectively. The value of globulin was shown to be significantly lower in healthy and mastitic buffaloes^43^ and cattle^65^ by other authors. This could be due to the invading microbe’s effects being neutralized by the creation of gamma-globulin, an antibody^46^.

There were no appreciably notable differences in serum ALT and ALP activations between the groups. Our findings on ALT were comparable to those obtained in cattle^40,42,65^ and in buffaloes^43^. Several publications have demonstrated that Red Sokoto goats had much higher ALT readings^45^which is in contradiction to the results described in the current study. Conversely^65^, verified a noteworthy rise in ALP serum levels in mastitic cow. Moreover^36^, observed a significant increase in ALT and ALP serum stages in mastitic camels.

### Antioxidants/oxidative stress markers

The present study found that, in comparison to the control group, mastitic ewes exhibited significantly higher levels of MDA and NO, and significantly lower levels of GSH, GPx, catalase, and SOD. Our results were consistent with those reported in camels and does by^36,65^respectively. These alterations could be attributed to the extreme requirement for high levels of GSH, SOD, catalase, and GPx activities for elevated degree oxidant damage caused by inflammatory reactions in the tissue of the mammary glands, or to an inadequate diet that influences the blood lipid peroxidation stage. Additionally, animal models with medical mastitis will exhibit elevated blood plasma levels of MDA^47^. The animal’s defensive systems against oxidants eventually multiplied. According to certain theories, mastitis is linked to higher levels of lipid peroxidation because of changes in the intracellular ratio of antioxidant device to free radicals^48^.

### Immune response

The current study found that mastitic sheep had significantly higher levels of haptoglobin, ceruloplasmin, amyloid A, and IgG than healthy sheep. This is consistent with the expanded ranges of APPs in^49^ in ewes with experimentally precipitated mastitis with Staphylococcus^38^, in gangrenous mastitis does^65^, in dairy cows with mastitis, and^36^ in mastitic camels. The release of proinflammatory cytokines such as TNF-α, which enhance the inflammatory process and stimulate neutrophil phagocytic activity, may be responsible for the elevated stages of APPs as a consequence of annoyance^67^.

### Correlations analysis

This is the first study to connect immunological and antioxidant marker serum profiles with gene expression in Barki ewes. Serum levels of SAA were negatively correlated with mRNA levels of *TNFα*, albumen serum levels were positively correlated with mRNA levels of *CAT*, and serum levels of NO were negatively correlated with mRNA levels of *OXSR1*. In addition, ALP, CP, and IgG serum levels were negatively correlated with mRNA levels of *Nrf2*, serum levels of AST were negatively correlated with mRNA levels of LTF, globulin serum levels were negatively correlated with mRNA levels of *INF-γ*. These correlations were found to be associated with various parameters. Our results are almost in line with those of^50^who discovered that in Egyptian buffalo cows with clinical endometritis, the mRNA levels of *CAT* and *GPX* had a positive correlation with the blood level of TNF-α (*r* = 1, *P* = 0.008 and *r* = 0.999, *P* = 0.034), respectively. Furthermore^27^, discovered that in Barki ewes with inflammatory postpartum diseases, there was a negative correlation between the mRNA levels of *CAT* and the serum levels of IL-1a, IL-1b, IL-6, IL-10, MDA, NO, CAT, GSH, and GPx. Therefore, changes in the mRNA level of the genes under investigation along with the serum profile of biochemical indicators might be used to accurately monitor the health condition of mastitic ewes. The latter criteria may be able to address the shortcomings of earlier research that relied on measuring isolated parameters in order to track the health of the animals.

Our work suggests that there is variation in genetic response between healthy and mastitic animals deciphered by genetic polymorphisms and transcript levels of immune and antioxidant indicators. Introducing new genetic markers and possible candidate genes for mastitis infection detection in Barki sheep offer potential chances to control the disease through selective breeding programs and marker assisted selection for mastitis resistant ewes. These could be used as trustworthy alternatives to predict and prevent the occurrence of mastitis in Barki sheep.

The present study showed some limitations, which should be considered in future studies. Firstly, the current investigation was carried out on a limited number of Barki ewes. As a result, more research on a large number of ewes is needed. Secondly, this investigation should be applied on different breeds of sheep for more an accurate health judgment. Thirdly, the present study did not use molecular or microbiological culture techniques to isolate or identify the pathogens that caused mastitis. Therefore, a further study is required to assess isolation or identification of the causative pathogens from mastitic milk samples either by using microbial culture or molecular methods. Fourthly, a limited number of genes related to immunity and antioxidant were examined. Thus, a wide range of factors has to be taken into account in subsequent research.

## Conclusion

The results herein confirm that there were profound immunological, antioxidant alterations associated with Barki sheep mastitis particularly blood AST, LDH, globulin, haptoglobin, ceruloplasmin, Amyloid A, IgG, MDA, NO, total protein, albumin, GSH, GPx, catalase and SOD. Our findings highlight the significance of SNPs in *IFN-γ*,* IL-4*,* TNF-α*,* MYD88*, *CCL5*,* TLR4*,* TLR9*,* LTF*,* PRLR*,* CAT*, *GPX1*, *Keap1*, *OXSR1*, *ATOX1*, *GST*, and *Nrf2* genes as genetic markers and predisposing factors for Barki sheep mastitis resistance/susceptibility. These results suggest that variations in these genes may serve as substitutes for these illnesses in Barki ewes. The difference in immune and antioxidant gene expression patterns between mastitis-resistant and non-resistant Barki ewes is a biomarker for monitoring sheep immunological state. This biomarker not only forecasts when a disease is most likely to appear, but it also creates a productive management plan to enhance health via appropriate breeding and immunization regimens.

## Materials and methods

### Animals and study design

A total of seventy multiparous ewes (fourth parity) were allocated into two equal size groups with clinical mastitis and healthy ewes taken as the control group with range of age 5–8 years (mean ± SD: 6.1 ± 1.1) and a range of body weight 34–47.5 kg (mean ± SD: 39.5 ± 4.3) at fourth lactation season. Sheep used in this study were obtained from the Desert Research Center’s Mariut Research Station in El-Amria, Alexandria, Egypt. Animals included in the study were in their 5th to 8th week of lactation period and were genetically related. Every animal in this research station has a comprehensive file with all of its information (ID, date of birth, age, sex, body weight, health status, schedule of vaccinations, course of treatment, sire ID, dam ID, and so on). The breeding system in the Mariut Research Station is selective breeding and usually starts in September and lasts for a period of 35 days (3 estrus cycles). All rams and ewes in the same flock are weighted and then selected for natural mating according to their visual appraisal of general health and conditions as well as their individual performance and parent offspring. The selected rams and ewes are divided into mating groups depending on their pedigree to avoid inbreeding. Ewes are joined in pens with single rams in groups of 20–25 ewes. After the mating period, ewes are separated from rams and kept as one group until lambing. The ewes were housed in semi-open shaded pens and fed on 750 gm concentrate feed mixture (CFM) plus 750 gm alfalfa hay/head/day, while water was always available ad libitum. The CFM was consisted of wheat bran (240 kg), soya bean (230 kg), corn (550 kg), sodium chloride (5 kg), calcium carbonate (10 kg), Premix (1 kg), Netro-Nill (0.5 kg) and Fylax (0.5 kg). The natural pasture (green herbage, grass and remnant of plant, berseem and darawa) was fed when available. The investigated ewes were subjected to clinical examination according to the standard protocols given previously^68^ and the findings were recorded simultaneously. The mammary glands of the Barki sheep were examined and clinical mastitis was defined based on physical examination of the gland through inspection and palpation^69^ and examination of expressed milk for abnormal color and consistency.

### Samples collection

#### Milk sample

According to^70^Teats were aseptically prepped prior to manual sample collection. After cleansing and drying the mammary teats, 70% ethanol was administered, and the first milk streams were thrown away. The milk samples (10–15 ml) from each ewe’s two mammary halves were then taken in sterile tubes, tagged, and sent directly to the lab for screening for somatic cell count (SCC) and California mastitis test (CMT). Somatic cells were counted electronically using a Bently Soma Count 150 (Bentley Instrument, Chaska, MN, USA). Three to five milliliters of sheep milk and an equivalent volume of CMT reagent were used for the CMT, which was provided by Taba Medical Pharma Company^®^. They were rapidly mixed by swirling or rotating. The response was rated based on the volume of gel that formed and the extent of color change^71^. Depending on the severity of the reaction, the CMT core was assigned a value of 0, + 1, +2, or + 3. Milk samples with CMT scores of 0 and 1 and SCC levels below 200,000/ml were considered healthy (*N* = 35), while milk samples with CMT scores of 2 or 3 and SCC levels above 200,000/ml were considered to have clinical mastitis (*N* = 35)^71^.

### Blood sample

Ten milliliters of blood were drawn from each sheep using a jugular vein puncture while the animals were completely secured. To obtain whole blood or serum, the samples were taken into vacutainer tubes with and without an anticoagulant (EDTA or sodium fluoride), respectively. While the blood in plain tubes was left at room temperature overnight and centrifuged for 15 min at 3000 rpm, the EDTA blood was utilized to extract DNA and RNA. For additional analysis of biochemical, inflammatory, and oxidative stress/antioxidant characteristics, the separated serum was kept at -80 °C.

### DNA extraction and polymerase chain reaction (PCR)

The genomic DNA was isolated from whole blood using the Gene JET whole blood genomic DNA extraction kit according to the manufacturer’s instructions (Thermo Scientific, Lithuania). PCR was used to amplify gene segments related to immunity (*IFN-γ*,* IL-4*,* TNF-α*,* MYD88*, *CCL5*,* TLR4*,* TLR9*,* LTF*, and *PRLR)* and antioxidant (*CAT*, *GPX1*, *Keap1*, *OXSR1*, *ATOX1*, *GST*, and *Nrf2*). The primer sequences were created in accordance with the *Ovis aries* nucleotide sequence for all investigated genes that was published in PubMed (https://www.ncbi.nlm.nih.gov/nuccore/?term=Sheep). A temperature cycler was used to prepare the polymerase chain reaction mixture, which had a final volume of 50 µl. In each reaction volume, there were 1 µl of each primer, 18 µl H_2_O (distilled water), 25 µl PCR master mix (2x) (Jena Bioscience, Germany), and 5 µl DNA. The reaction mixture was first subjected to a denaturation temperature of 94 °C for eight minutes. The cycling technique consisted of thirty cycles of denaturation for one minute at 94 °C, annealing temperatures for 45 s (as shown in Table 6), extension for 45 s at 72 °C, and a final extension for eight minutes at 72 °C.

**Table 6:** Forward and reverse primer sequence, length of PCR product and annealing temperature for immune and antioxidant genes used in PCR-DNA sequencing.

Gene	Forward	Reverse	Annealing temperature (°C)	Length of PCR product (bp)	Reference
*IFN-γ*	5′-CCCAGATGTAGCTAAGGGTGG − 3′	5′-CTCTCCGGCCTCGAAAGAGATTC-3′	60	365	Current study
*IL-4*	5′-TGCTTACTGGTATGTACCAGC − 3′	5′-CACAGAACAGGTCTTGCTTGCCA − 3′	62	285	Current study
*TNF-α*	5′-CTGCCGGAATACCTGGACTATG-3′	5′-TTCCAGTCCTTGGTGATGGTTG-3′	58	273	Current study
*MYD88*	5′-ATGACTGAAGGAGTCCCCAGCG-3′	5′-CACCATCCGACGGCACCTCTTC-3′	64	660	Current study
*CCL5*	5′-AGCTGCAGAGGATCAGCACGTG-3′	5′-TCAGGTTCAAGGCGTCCTCCAC-3′	62	350	Current study
*TLR4*	5′-TGCAGATGGTATGGTTGACCAG-3′	5′-AATGCTTCAGGTTGGTTGTCC-3′	60	256	Current study
*TLR9*	5′-TTCGTGGACCTGTCGGACAACCG-3′	5′-CTGGCTGTTGTAGCTGAGGTC-3′	62	414	Current study
*LTF*	5′-GATATCCTTTTCATTGGCAAATG-3′	5′-TTGTGGATCAGCTTGACTGTACA-3′	64	299	Current study
*PRLR*	5′-AAGGGCAAGTCCGAAGAACTTCT-3′	5′-CTATGGCAGGGCTGGCGGGGCCT-3′	62	891	Current study
*CAT*	5′-CAGCCAGCGACCAGATGAAAC-3′	5′-TCCTCTTTCCAATATGCTCAAAC-3′	62	300	Current study
*GPX1*	5′-GATGAATGACCTGCAGCGGCG-3′	5′-TCTCCCGAAGGAAGGCGAAGA-3′	58	221	Current study
*KEAP1*	5′-CAACAGCGAAAGTCAGGCGGGA-3′	5′-TGAAGGTGCGGTTGCCATGCTG-3′	64	360	Current study
*OXSR1*	5′-GGGTTTGGAATACCTGCAT-3′	5′-GGGTACTTATGATAAGGAGCTG-3′	58	357	Current study
*ATOX1*	5′-GGAGGCGTAGTCACCGCCGCAG-3′	5′-TCGCCAGCAAAATCGGCTTGACT-3′	58	433	Current study
*GST*	5′-GTGGGCAAGCCCAAGCTGCACTA-3′	5′-AACCAGGTGGATGTCAGCCTTGC-3′	62	480	Current study
*Nrf2*	5′-CACTGAACACAACAAGTCCAAGC-3′	5′-TCATCTCTTGTGAGGTGAGCC-3′	64	340	Current study

*IFN-γ* interferon gamma, *IL-4* iterleukin-4, *TNF-α* tumor necrosis factor-alpha, *MYD88* myeloid differentiation primary response 88, *CCL5* chemokine (C-C motif) ligand 5, *TLR4* toll-like receptor 4, *TLR9* toll-like receptor 9, *LTF* lactoferrin, *PRLR* prolactin receptor, *CAT* catalase, *GPX1* glutathione peroxidase 1, *KEAP1* Kelch-like ECH-associated protein 1, *OXSR1* oxidative stress responsive kinase 1, *ATOX1* antioxidant 1 copper chaperone 1, *GST* glutathione S transferase, *Nrf2* nuclear factor-erythroid factor 2-related factor.

### Polymorphism detection

#### DNA sequencing

PCR purification was carried out using a kit provided by the manufacturer (Jena Bioscience # pp-201×s/Germany) to produce PCR products with targeted bands of the expected sizes, as stated by Boom et al. (1990)^72^. Utilizing the Nanodrop (Uv-Vis spectrophotometer Q5000/USA) for PCR product measurement allowed for the achievement of excellent yields, adequate concentrations, and purity of the PCR products^73^.

PCR products with target band were sent for forward and reverse DNA sequencing using an ABI 3730XL DNA sequencer (Applied Biosystem, USA), that is based on the enzymatic chain terminator technique developed by Sanger et al. 1977 ^74^, to detect single nucleotide polymorphisms (SNPs) in genes investigated in control and mastitis-affected ewes.

#### Analysis of nucleotide and amino acid sequence among healthy and mastitic Ewes

The DNA sequencing results were analysed using the software packages Blast 2.0 and Chroma 1.45^75^. Variations between the PCR products of the genes under study and reference sequences found in GenBank were categorized as SNPs. In accordance with data alignment from DNA sequencing, the MEGA4 software tool was used to alter the amino acid sequence of the genes under inquiry in enrolled animals^57^.

### Total RNA extraction, reverse transcription and quantitative real time PCR

Following the manufacturer’s instructions, total RNA was extracted from sheep blood using Trizol reagent (RNeasy Mini Ki, Catalogue no. 74104). The synthesis of the cDNA for each sample was done in accordance with the manufacture methodology (Thermo Fisher, Catalogue no. EP0441). Using qRT-PCR (Agilent MX3005P, CA, USA) and SYBR Green PCR Master Mix (2x SensiFastTM SYBR, Bioline, CAT No: Bio-98002), an analysis was conducted on the pattern of gene expression pertaining to immunity and antioxidant genes.

SYBR Green PCR Master Mix (Quantitect SYBR green PCR kit, Catalogue no. 204141) was used to perform qRT-PCR for the relative measurement of the mRNA level. The primer sequences were created based on the *Ovis aries* published sequence in PubMed, as indicated in Table 7. As a constitutive control for normalization, the housekeeping gene *ß-actin* was employed. In a total volume of 25 µl, the reaction mixture was composed of 3 µl of total RNA, 4 µl of 5x Trans Amp buffer, 0.25 µl of reverse transcriptase, 0.5 µl of each primer, 12.5 µl of 2x Quantitect SYBR green PCR master mix, and 8.25 µl of RNase-free water.

**Table 7:** Oligonucleotide primers sequence, accession number, annealing temperature and PCR product size of immune and antioxidant genes used in real time PCR.

Gene	Primer	Product length (bp)	Annealing Temperature (°C)	Accession number	Source
*IFN-γ*	F5′-GATGATCTGCAGATCCAGCGCA-3 R5′-AGAGATTCTGACTTCTCTTCCG-3′	106	60	AY575608.1	Current study
*IL-4*	F5′-GGAATTGAGCTTAGGCGTAT-3′ R5′-TCTTGCTTGCCAGGCTGCTGA-3′	94	62	AY575607.1	Current study
*TNF-α*	F5′-ACTATGCCGAGTCTGGGCAGG-3′ R5′-GCTTGGAGCCCAGCCCTGAG-3′	164	58	AY513771.1	Current study
*MYD88*	F5′-CTGAAGCAGCAGCAGGAGGCAT-3′ R5′-GTCGCGAATAGTGATGCCTGCCA-3′	102	58	NM_001166183.1	Current study
*CCL5*	F5′-′CACCAGCAGCAAGTGCTCCATG-3′ R5′-CACTTCTTCTCTGGGTTGGCGCA-3′	84	60	XM_005693201.3	Current study
*TLR4*	F5′-TAGGAAGTCTACAAGCCCTTCT-3′ R5′-CAGGAAACTTATCAAAGTCACA-3′	81	62	MW201968.1	Current study
*TLR9*	F5′-AGCAGGAGATGTTTACCCGCCTC-3′ R5′-AGCACTCGCAGGCCGGTCAGCG-3′	115	64	HQ717159.1	Current study
*LTF*	F5′-GTGGTGTCTCGGAGCGATAG-3′ R5′-CATACGTTGGTCTGCCTCCA-3′	184	62	AY792499.1	Current study
*PRLR*	F5′-CATGGCATGGCCACTGCTCCA-3′ R5′-CTTCGAGTACTCCTTGCTGGTT-3′	160	60	FJ901299.1	Current study
*CAT*	F5′-CAGTAGGAGACAAACTCAATG-3′ R5′-ACGACTCTCTCAGGAATTCTC-3′	121	62	GQ204786.1	Current study
*GPX1*	F5′-CGAGGAGATCCTGAATTGCCTGA-3′ R5′-ACCTCGCACTTTTCGAAGAGC-3′	54	60	JF728302.1	Current study
*KEAP1*	F5′-TGAGAGTATCGGAGGCTACGCA-3′ R5′-CGCTAGGCCTGGGTTCCGGCT-3′	95	62	XM_027969637.2	Current study
*OXSR1*	F5′-ACAGATTCACAGAGATGTGAAAG-3′ R5′-CCACGAACCTGTTCCATAACT-3′	186	58	XM_027958018.2	Current study
*ATOX1*	F5′-GCAGCCACCACCTCCTCCTCAA-3′ R5′-GTGCTCAGAGTTGATGCAGAC-3′	122	58	XM_005683194.3	Current study
*GST*	F5′-TGGCTGCAGCCGGAGTGGAGTT-3′ R5′-TGGCAACGTAGTTGAGAATGGC-3′	162	64	AJ238319.1	Current study
*Nrf2*	F5′-TCACAAGGGAACAGCACTGCAG-3′ R5′-AGCCTCCAAGCGGCTTGAATG-3′	129	64	NM_001314327.1	Current study
*ß. actin*	F5′-GCAAAGACCTCTACGCCAAC-3′ R5′-TGATCTTCATCGTGCTGGGT-3′	114	60	NM_001009784.3	Current study

*IFN-γ* interferon gamma, *IL-4* iterleukin-4, *TNF-α* tumor necrosis factor-alpha, *MYD88* myeloid differentiation primary response 88, *CCL5* chemokine (C-C motif) ligand 5, *TLR4* toll-like receptor 4, *TLR9* toll-like receptor 9, *LTF* lactoferrin, *PRLR* prolactin receptor, *CAT* catalase, *GPX1* glutathione peroxidase 1, *KEAP1* Kelch-like ECH-associated protein 1, *OXSR1* oxidative stress responsive kinase 1, *ATOX1* antioxidant 1 copper chaperone 1, *GST* glutathione S transferase, *Nrf2* nuclear factor-erythroid factor 2-related factor.

The following procedure was carried out once the final reaction mixture was put in a heat cycler: Reverse transcription was conducted for 30 min at 50 °C, initial denaturation for 10 min at 94 °C, 40 cycles of 94 °C for 15 s, annealing temperatures in accordance with Table 7, and 30 s at 72 °C. The specificity of the PCR product was confirmed by a melting curve study once the amplification process was complete. The 2^−ΔΔCt^ technique was utilized to calculate the relative expression of each gene per sample in relation to the *ß-actin* gene^41^.

### Blood analysis

#### Biochemical markers

The serum activities of alanine aminotransferase (ALT, catalog No.; AL146), aspartate aminotransferase (AST, catalog No.; AS101) (Randox, UK), alkaline phosphatase (ALP, catalog No.; A504-150) (Teco Diagnostics, USA), and lactate dehydrogenase (Spinreact, Spain) (LDH, catalog No.; TK41214) were assessed according to manufacturer’s protocol in the enclosed pamphlets. Additionally, serum levels of total protein (Catalog No.; SB-0250-500) and albumin (Catalog No.; SB- 028–500) (Stanbio Laboratory, USA).

#### Inflammatory markers

Haptoglobulin (HP, Catalog No.; ACN 228), ceruloplasnim (CP, Catalog No.; CHN 807), and immunoglobulin G (IgG, REF; 03507432) (Cobas Co., USA) were estimated according to the standard protocol of their specific pamphlets. Serum amyloide A was also evaluated using ELISA kits (SAA, Catalog No.; EHSAA1) obtained from Thermo Fisher Scientific according to its manufacturer’s instructions. All parameters were measured spectrophotometrically using 5010 Photometer (BM Co. Germany).

#### Oxidative stress/antioxidant parameters

The serum levels of malondialdehyde (MDA, Catalog No.; MD 25 29), nitric oxide (NO, Catalog No.; NO 25 33), glutathione (GSH, Catalog No.; GR 25 11), glutathione peroxidase (GPx, Catalog No.; GP 25 24), catalase (Catalog No.; CA 25 17) and superoxide dismutase (SOD, Catalog No.; SD 25 21) were estimated spectrophotometrically using commercial test kits obtained from Biodiagnostics Co., (Cairo, Egypt).

### Statistical analysis

Independent Samples t-Test (SPSS software program, version 20, USA) was used to determine the differences in the biochemical parameters and gene expression of tested enzymes among animal of different disease status (mastitis and non mastitic groups). This was utilized to assess whether or not the biochemical markers and SNPs found were significantly linked to the presence of mastitis. The values were expressed as mean ± SD. While we utilized Pearson’s simple correlation test to determine the direction and significance of the association between the biochemical parameters and gene expression of tested enzymes in diseased group only. Correlation coefficient (r) and P value were considered. A difference was considered significant at *P* < 0.05. We solely used the gene expression approach and did not genotype the genes under study; hence genotype-phenotype correlation analyses were not necessary.

Difference in the frequencies of each gene SNPs between mastitic and healthy ewes was statistically evaluated using Chi-square test to compare the distribution of the identified SNPs between the two groups using SPSS version 23, USA.

For each ewe, a mean SNP score was calculated per gene by averaging the numeric values of all SNPs assigned to that gene. This average gene score was intended to represent the overall genetic variation of each gene within an individual. A Linear Discriminant Analysis (LDA) was conducted to determine whether gene-level SNP averages could differentiate between mastitic and healthy ewes. The 16 gene average scores served as predictor variables, and the health status (mastitic vs. healthy) was the grouping variable Statistical significance was set at *p* < 0.05.

## Electronic supplementary material

Below is the link to the electronic supplementary material.

Supplementary Material 1

## Data Availability

The datasets for sequence generated and/or analyzed during the current study are available in the GenBank with accession numbers gb|PP567235|, gb|PP567236|, gb|PP567237|, gb|PP567238|, gb|PP567239|, gb|PP567240|, gb|OR900038|, gb|OR900039|, gb|OR900040|, gb|PP575806|, gb|PP575807|, gb|PP575808|, gb|PP575809|, gb|PP575810|, gb|PP575811|, gb|PP575812|, gb|PP575813|, gb|PP575814|, gb|PP575815|, gb|PP575816|, gb|PP575817|, gb|PP575818|, gb|PP575819|, gb|PP596786|, gb|PP596787|, gb|PP596788|, gb|PP596789|, gb|PP596790|, gb|PP596791|, gb|PP596792|, gb|PP596793|, gb|PP596794|, gb|PP596795|.

## Abbreviations

IFN-γ: Interferon gamma
IL-4: Iterleukin-4
TNF-α: Tumor necrosis factor-alpha
MYD88: Myeloid differentiation primary response 88
CCL5: Chemokine (C-C motif) ligand 5
TLR4: Toll-like receptor 4
TLR9: Toll-like receptor 9
LTF: Lactoferrin
PRLR: Prolactin receptor
CAT: Catalase
GPX1: Glutathione peroxidase 1
KEAP1: Kelch-like ECH-associated protein 1
OXSR1: Oxidative stress responsive kinase 1
ATOX1: Antioxidant 1 copper chaperone 1
GST: Glutathione S transferase
Nrf2: Nuclear factor-erythroid factor 2-related factor
A: Alanine
C: Cisteine
D: Aspartic acid
E: Glutamic acid
F: Phenylalanine
G: Glycine
H: Histidine
I: Isoleucine
K: Lysine
L: Leucine
M: Methionine
N: Asparagine
P: Proline
Q: Glutamine
R: Argnine
S: Serine
T: Threonine
V: Valine
W: Tryptophan
Y: Tyrosine
ALT: Alanine aminotransferase
AST: Aspartate aminotransferase
ALP: Alkaline phosphatase
LDH: Lactate dehydrogenase
IgG: Immunoglobulin G
MDA: Malondialdehyde
NO: Nitric oxide
GSH: Reduced glutathione
GPx: Glutathione peroxidase
SOD: Superoxide dismutase
