# Supplementary material for: Evaluating gene expression and biomarkers for mastitis resistance in Barki sheep
Source: Sci Rep. 2025 Jun 20;15:20127. doi: 10.1038/s41598-025-04515-1 (PMC12181237; doi:10.1038/s41598-025-04515-1)
Supplement: Supplementary file 1 — Supplementary Material 1 [file 41598_2025_4515_MOESM1_ESM.pdf]

```
AY575608.1      CCCAGATGTAGCTAAGGGTGGGCCTCTTTTCTCAGAAATTCTGAAGAATTGGAAAGAGGA      60
H               CCCAGATGTAGCTAAGGGTGGGCCTCTTTTCTCAGAAATTTTGAAGAATTGGAAAGAGGA      60
M               CCCAGATGTAGCTAAGGGTGGGCCTCTTTTCTCAGAAATTCTGAAGAATTGGAAAGAGGA      60
                **************************************** *******************

AY575608.1      GAGCGACAAAAAGATTATTCAGAGCCAAATTGTCTCTTTCTACTTCAAACTCTTTGAAAA      120
H               GAGCGACAAAAAGATTATTCAGAGCCAAATTGTCTCTTTCTACTTCAAACTCTTTGAAAA      120
M               GAGCGACAAAAAGATTATTCAGAGCCAAATTGTCTCCTTCTACTTCAAACTCTTTGAAAA      120
                ************************************ ***********************

AY575608.1      CCTCAAAGATAACCAGGTCATTCAAAGGAGCATGGATATCATCAAGCAAGACATGTTTCA      180
H               CCTCAAAGATAACCAGGTCATTCAAAGGAGCATGGATATCATCAAGCAAGACATGTTTCA      180
M               CCTCAAAGATAACCAGGTCATTCAAAGGAGCATGGATATCATCAAGCAAGACATGTTTCA      180
                ************************************************************

AY575608.1      GAAGTTCTTGAATGGCAGCTCTGAGAAACTGGAGGACTTCAAAAGGCTGATTCAAATTCC      240
H               GAAGTTCTTGAACGGCAGCTCTGAGAAACTGGAGGACTTCAAAAGGCTGATTCAAATTCC      240
M               GAAGTTCTTGAATGGCAGCTCTGAGAAACTGGAGGACTTCAAAAGGCTGATTCAAATTCC      240
                ************ ***********************************************

AY575608.1      AGTGGATGATCTGCAGATCCAGCGCAAAGCCATCAATGAACTCATCAAGGTGATGAATGA      300
H               AGTGGATGATCTGCAGATCCAGCGCAAAGCCATCAATGAACTCATCAAGGTGATGAATGA      300
M               GGTGGATGATCTGCAGATCCAGCGCAAAGCCATCAATGAACTCATCAAGGTGATGAATGA      300
                 ***********************************************************

AY575608.1      CCTGTCGCCAAAATCTAACCTCAGAAAGCGGAAGAGAAGTCAGAATCTCTTTCGAGGCCG      360
H               CCTGTCGCCAAAATCTAACCTCAGAAAGCGGAAGAGAAGTCAGAATCTCTTTCGAGGCCG      360
M               CCTGTCGCCAAAATCTAACCTCAGAAAGCGGAAGAGAAGTCAGAATCTCTTTCGAGGCCG      360
                ************************************************************

AY575608.1      GAGAG      365
H               GAGAG      365
M               GAGAG      365
                *****
```

Figure S1: Representative sequence alignment of the *IFN-γ* gene (365 bp) between mastitic (M) and healthy (H) ewes compared to reference GenBank accession number (AY575608.1).

```
AY575607.1      TGCTTACTGGTATGTACCAGCCACTTCGTCCATGGACACAAGTGTGATATTACCTTAGAA       60
H               TGCTTACTGGTATGTACCAGCCACTTCGTCCATGGACACATGTGTGATATTACCTTAGAA       60
M               TGCTTACTGGTATGTACCAGCCACTTCGTCCATGGACACAAGTGTGATATTACCTTAGAA       60
                **************************************** *******************

AY575607.1      GAGATCATCAAAACGCTGAACATCCTCACATCGAGAAAGAATTCATGCATGGAGCTGCCT       120
H               GAGATCATCAAAACGCTGAACGTCCTCACATCGAGAAAGAATTCATGCATGGAGCTGCCT       120
M               GAGATCATCAAAACGCTGAACATCCTCACATCGAGAAAGAATTCATGCATGGAGCTGCCT       120
                ******************** ***************************************

AY575607.1      GTAGCAGACGTCTTTGCTGCCCCAAAGAACGCAACTGAGAAGGAAACCTTCTGCAGGGCT       180
H               GTAGCAGACGTCTTTGCTGCCCCAAAGAACGCAACTGAGAAGGAAACCTTCTGCAGGGCT       180
M               GTAGCAGACGTCTTTGCTGCCCCAAAGAACGCAACTGAGAAGGAAACCTTCTGCAGGGCT       180
                ************************************************************

AY575607.1      GGAATTGAGCTTAGGCGTATCTACAGGAGCCACATGTGCTTGAACAAATTCCTGGGCGGA       240
H               GGAATTGAGCTTAGGCGTATCTACAGGAGCCACATGTGCTTGAACAAATTCCTGGGCGGA       240
M               GGAATTGAGCTTAGGCGTATCTACAGGAGCCACATGTGCTTGAACAAATTCCTGGGCGGA       240
                ************************************************************

AY575607.1      CTTGACAGGAATCTCAGCAGCCTGGCAAGCAAGACCTGTTCTGTG    285
H               CTTGACAGGAATCTCAGCAGCCTGGCAAGCAAGACCTGTTCTGTG    285
M               CTTGACAGGAATCTCAGCAGCCTGGCAAGCAAGACCTGTTCTGTG    285
                *********************************************
```

Figure S2: Representative sequence alignment of the *IL-4* gene (285 bp) between mastitic (M) and healthy (H) ewes compared to reference GenBank accession number (AY575607.1).

```
AY513771.1      CTGCCGGAATACCTGGACTATGCCGAGTCTGGGCAGGTCTACTTTGGGATCATCGCCCTG      60
H               CTGCCGGAATACCTGGACTATGCCGAGTCTGGGCAGGTCTACTTTGGGATCATCGCCCTG      60
M               CTGCCGGAATACCTGGACTATGCCGAGTCTGGGCAGGTCTACTTTGGGATCATCGCCCTG      60
                ************************************************************

AY513771.1      TGAGGGCGCAGGACATGCATCCTCTCCCACCTCAGTTACCTTATTATTTACTCCTTCAGA      120
H               TGAGGGCGCAGGACATGCATCCTCTCCCACCTCAGTTACCTTATTATTTACTCCTTCAGA      120
M               TGAGGGCGCAGGACATGCATCCTCTCCCACCTCAGTTACCTTATTATTTACTCCTTCAGA      120
                ************************************************************

AY513771.1      CCCTCCTCATCCCCTTCTGGTTTAGAAAGGGAATTAGGGGCTCAGGGCTGGGCTCCAAGC      180
H               CCCTCCTCATCCCCTTCTGGTTTAGAAAGGGAATTAGGGGCTCAGGGCTGGGCTCCAAGC      180
M               CCCTCCTCATCCCCTTCTGGTTTAGAAAGGGAATTAGGGGCTCAGGGCTGGGCTCCAAGC      180
                ************************************************************

AY513771.1      GTCCAACTTTAAACAACAGCTGCACTTAGAAATTAGGGATGTAGGGAAGTGAGGCCTGGA      240
H               GTCCAACTTTAAACAACAGCTGCACTTAGAAATTAGGGATGTAGGGAAGTGAGGCCTGGA      240
M               GTCCAACTTTAAACAACAGCTGCACTTAGAAATTAGGGATGTAGGGAAGTGAGGCCTGGA      240
                ************************************************************

AY513771.1      CAATGGGCCACCAACCATCACCAAGGACTGGAA      273
H               CAATGGGCCACCAACCATCACCAAGGACTGGAA      273
M               CAACGGGCCACCAACCATCACCAAGGACTGGAA      273
                *** *****************************
```

Figure S3: Representative sequence alignment of the *TNF-α* gene (273 bp) between mastitic (M) and healthy (H) ewes compared to reference GenBank accession number (AY513771.1).

```
M              ATGACTGAAGGAGTCCCCAGCGCGGGGTCCGCGCTCCCCACGCCTGCCATGTTCTCCCTG     60
MK986727.1     ATGACTGAAGGAGTCCCCAGCGCGGGGTCCGCGCTCCCCACGCCTGCCATGTCCTCCCTG     60
H              ATGACTGAAGGAGTCCCCAGCGCGGGGTCCGCGCTCCCCACGCCTGCCATGTCCTCCCTG     60
               **************************************************** *******

M              CCGCTGGCAGCGCTCAACGTGCGAGTGCGGCGCCGCCTGTCGCTCTTCCTAAACGTGCGG     120
MK986727.1     CCGCTGGCAGCGCTCAACGTGCGAGTGCGGCGCCGCCTGTCGCTCTTCCTAAACGTGCGG     120
H              CCGCTGGCAGCGCTCAACGTGCGAGTGCGGCGCCGCCTGTCGCTCTTCCTAAACGTGCGG     120
               ************************************************************

M              GCGCCGGTGGCGGCCGACTGGACCGTGCTGGCGGAGGCGATGGACTTCGAGTACTTGGAG     180
MK986727.1     GCGCCGGTGGCGGCCGACTGGACCGTGCTGGCGGAGGCGATGGACTTCGAGTACTTGGAG     180
H              GCGCCGGTGGCGGCCGACTGGACCGTGCTGGCGGAGGCGATGGACTTCGAGTACTTGGAG     180
               ************************************************************

M              ATCCAGCAGCTGGAGAAGTACGCCGACCCCACGAGCAGGCTGCTGGACGACTGGCAGCGA     240
MK986727.1     ATCCAGCAGCTGGAGAAGTACGCCGACCCCACGAGCAAGCTGCTGGACGACTGGCAGCGA     240
H              ATCCAGCAGCTGGAGAAGTACGCCGACCCCACGAGCAAGCTGCTGGACGACTGGCAGCGA     240
               ************************************* **********************

M              CGTCCGGGCGCCTCAGTGGGCCGCCTGCTCGAGCTGCTCGCCAAGCTCGGCCGCGAGGAC     300
MK986727.1     CGTCCGGGCGCCTCAATGGGCCGCCTGCTCGAGCTGCTCGCCAAGCTCGGCCGCGAGGAC     300
H              CGTCCGGGCGCCTCAATGGGCCGCCTGCTCGAGCTGCTCGCCAAGCTCGGCCGCGAGGAC     300
               *************** ********************************************

M              GTGCTGATGGAACTGGGACCCAGCATCGAGGAGGACTGCCAAAAGTATATTCTGAAGCAG     360
MK986727.1     GTGCTGATGGAACTGGGACCCAGCATCGAGGAGGACTGCCAAAAGTATATTCTGAAGCAG     360
H              GTGCTGATGGAACTGGGACCCAGCATCGAGGAGGACTGCCAGAAGTATATTCTGAAGCAG     360
               ***************************************** ******************

M              CAGCAGGAGGCATCTGAGAAGCCTTTACAGGTGGACTCTATAGACAGCAGCATCCCTCGG     420
MK986727.1     CAGCAGGAGGCATCTGAGAAGCCTTTACAGGTGGACTCTATAGACAGCAGCATCCCTCGG     420
H              CAGCAGGAGGCATCTGAGAAGCCTTTACAGGTGGACTCTATAGACAGCAGCATCCCTCGG     420
               ************************************************************

M              ATAAATGACATGGCAGGCATCACTATTCGCGACGACCACCTAGGGCAAAAGCCTGAGTAT     480
MK986727.1     ATAAATGACATGGCAGGCATCACTATTCGCGACGACCCCCTAGGGCAAAAGCCTGAGTAT     480
H              ATAAATGACATGGCAGGCATCACTATTCGCGACGACCCCCCAGGGCAAAAGCCTGAGTAT     480
               ************************************* ** *******************

M              TTTGATGCCTTCATCTGCTACTGCCCCAGCGATATTGAGTTTGTCCATGAGATGATCCGG     540
MK986727.1     TTTGATGCCTTCATCTGCTACTGCCCCAGCGATATTGAGTTTGTCCATGAGATGATCCGG     540
H              TTTGATGCCTTCATCTGCTACTGCCCCAGCGATATTGAGTTTGTCCACGAGATGATCCGG     540
               *********************************************** ************

M              CAGCTGGAACAGACAAACTATCGGCTGAAATTGTGCGTGTCTGACCGTGACGTCCTGCCT     600
MK986727.1     CAGCTGGAACAGACAAACTATCGGCTGAAGTTGTGCGTGTCTGACCGTGACGTCCTGCCT     600
H              CAGCTGGAACAGACAAACTATCGGCTGAAGTTGTGCGTGTCTGACCGTGACGTCCTGCCT     600
               **************************** *******************************

M              GGCACCTGTGTCTGGTCCATCGCCAGTGAACTCATTGAGAAGAGGTGCCGTCGGATGGTG     660
MK986727.1     GGCACCTGTGTCTGGTCCATCGCCAGTGAACTCATTGAGAAGAGGTGCCGTCGGATGGTG     660
H              GGCACCTGTGTCTGGTCCATCGCCAGTGAACTCATTGAGAAGAGGTGCCGTCGGATGGTG     660
               ************************************************************
```

Figure S4: Representative sequence alignment of the *MYD88* gene (660 bp) between mastitic (M) and healthy (H) ewes compared to reference GenBank accession number (MK986727.1).

```
XM_005693201.3      AGCTGCAGAGGATCAGCACGTGGATCGCCCCAGCCTCTGACCACAGCTACCATGAAGGTC   60
H                   AGCTGCAGAGGATCAGCACGTGGATCGCCCCAGCCTCTGACCACAGCTACCATGAAGGTC   60
M                   AGCTGCAGAGGATCAGCACGTGGATCGCCCCAGCCTCTGACCACAGCTACCATGAAGGTC   60
                    ************************************************************

XM_005693201.3      TCTTCCGCTGCCCTCGCTGTCCTCCTGATGGCGGCCGCCCTCTGCGCTCCTGCGTCTGCC   120
H                   TCTTCCGCTGCCCTCGCTGTCCTCCTGATGGCGGCCGCCCTCTGCGCTCCTGCGTCTGCC   120
M                   TTCTCCGCTGCCCTCGCTGTCCTCCTGATGGCGGCCGCCCTCTGCGCTCCTGCGTCTGCC   120
                    *  *********************************************************

XM_005693201.3      TCCCCATATGCCTCGGACACCACCCCCTGCTGTTTTGCCTACCTCTCCCGCCCGCTGCCC   180
H                   TCCCCATATGCCTCGGACACCACCCCCTGCTGTTTTGCCTACCTCTCCCGCCCGCTGCCC   180
M                   TCCCCATATGCCTCGGACACCACGCCCTGCTGCTTTGCCTACCTCTCCCGCCCGCTGCCC   180
                    *********************** ******** ***************************

XM_005693201.3      CGCACCCACGTCCAGGAATATTTCTACACCAGCAGCAAGTGCTCCATGGCAGCAGTTGTC   240
H                   CGCAACCACGTCCAGGAATATTTCTACACCAGCAGCAAGTGCTCCATGGCAGCAGTTGTC   240
M                   CGCACCCACGTCCAGGAATATTTCTACACCAGCAGCAAGTGCTCCATGGCAGCAGTTGTC   240
                    **** *******************************************************

XM_005693201.3      TTTATCACCAGGAAGAACCGTCAGGTGTGCGCCAACCCAGAGAAGAAGTGGGTGCGAGAG   300
H                   TTTATCACCAGGAAGAACCGTCAGGTGTGCGCCAACCCAGAGAAGAAGTGGGTGCGAGAG   300
M                   TTTATCACCAGGAAGAACCGTCAGGTGTGCGCCAACCCAGAGAAGAAGTGGGTTCGAGAG   300
                    ***************************************************** ******

XM_005693201.3      TACATCAACAGTTTGGAGTTGAGCTAGGGTGGAGGACGCCTTGAACCTGA   350
H                   TACATCAACAGTTTGGAGTTGAGCTAGGGTGGAGGACGCCTTGAACCTGA   350
M                   TACATCAACAGTTTGGAGTTGAGCTAGGGTGGAGGACGCCTTGAACCTGA   350
                    **************************************************
```

Figure S5: Representative sequence alignment of the *CCL5* gene (350 bp) between mastitic (M) and healthy (H) ewes compared to reference GenBank accession number (XM_005693201.3).

```
MW201966.1      TCTTTAGGAAGTCTACAAGCCCTTCTTAAAGATTTTAGATGGCAACACTTAGAAATGATT       60
H               TCTTTAGGAAGTCTACAAGCCCTTCTTAAAGATTTTAGATGGCAACACTTAGAAATGATT       60
M               TCTTTAGGAAGTCTACAAGCCCTTCTTAAAGATTTTAGATGGCAACACTTAGAAATGATT       60
                ************************************************************

MW201966.1      AACTGTGACTTTGATAAGTTTCCTGCACTGAAGCTCCGTTCTCTCAAAAAGTTTGTTTTC       120
H               AACTGTGACTTTGATAAGTTTCCTGCACTGAAGCTCCGTTCTCTCAAAAAGTTTGTTTTC       120
M               AACTGTGACTTTGATAAGTTTCCTGCACTGGAGCTCCGTTCTCTCAAAAAGTTTGTTTTC       120
                ****************************** *****************************

MW201966.1      ACAGACAACAAAGATGTAAGCAGTTTTACTAAGACTGAGCTACCAAGCCTTCAGTATCTA       180
H               ACAGACAACAAAGATGTAAGCACTTTTACTAAGACTGAGCTACCAAGCCTTCAGTATCTA       180
M               ACAGACAACAAAGGTGTAAGCAGTTTTACTAAGACTGAGCTACCAAGCCTTCAGTATTTA       180
                ************* ******** ********************************** **

MW201966.1      GATCTCAAAAGAAATCACTTGAGTTTCAAGACCTGCTGTTCTCACACTTATTTTGGGACA       240
H               GATCTCAAAAGAAATCACTTGAGTTTCAAGACCTGCTGTTCTCACACTTATTTTGGGACA       240
M               GATCTCAAAAGAAATCACTTGAGTTTCAAGACCTGCTGTTCTCACACTGATTTTGGGACA       240
                ************************************************ ***********

MW201966.1      ACCAACCTGAAGCATT     256
H               ACCAACCTGAAGCATT     256
M               ACCAACCTGAAGCATT     256
                ****************
```

Figure S6: Representative sequence alignment of the *TLR4* gene (256 bp) between mastitic (M) and healthy (H) ewes compared to reference GenBank accession number (MW201966.1).

```
JN377803.1      TTCGTGGACCTGTCGGACAACCGCATCAGCGGAGCTGCGAGGCCGGTGGCCGCCCTCGGG       60
H               TTCGTGGACCTGTCGGACAACCGCATCAGCGGAGCTGCGAGGCCGGTGGCCGCCCTCGGG       60
M               TTCGTGGACCTGTCGGACAACCGCATCAGCGGAGCTGCGAGGCCGGTGGCCGCCCTCGGG       60
                ************************************************************

JN377803.1      GAGGTGGACAGCGGGGTGGAAGTCTGGCGGTGGCCCAGGGGCCTCGCTCCAGGCCCGCTG       120
H               GAGGTGGACAGTGGGGTGGAAGTCTGGCAGTGGCCCAGGGGCCTCGCTCCAGGCCCGCTG       120
M               GAGGTGGACAGCGGGGTGGAAGTCTGGCGGTGGCCCAGGGGCCTCGCTCCAGGCCCGCTG       120
                *********** **************** *******************************

JN377803.1      GCCGCCGTCAGCTCAAAGGACTTCATGCCAAGCTGCAACCTCAACTTCACCTTGGACCTG       180
H               GCCGCCGTCAGCGCAAAGGACTTCATGCCAAGCTGCAACCTCAACTTCACCTTGGACCTG       180
M               GCCGCCGTCAGCTCAAAGGACTTCATGCCAAGCTGCAACCTCAACTTCACCTTGGACCTG       180
                ************ ***********************************************

JN377803.1      TCACGGAACAACCTGGTGACGATCCAGCAGGAGATGTTTACCCGCCTCTCCCGCCTCCAG       240
H               TCACGGAACAACCTGGTGACGATCCAGCAGGAGATGTTTACCCGCCTCTCCCGCCTCCAG       240
M               TCACGGAACAACCTGGTGACAATCCAGCAGGAGATGTTTACCCGCCTCTCCCGCCTCCAG       240
                ******************** ***************************************

JN377803.1      TGCCTGCGCCTGAGCCACAACAGCATCTCGCAGGCGGTTAATGGCTCGCAGTTCGTGCCG       300
H               TGCCTGCGCCTGAGCCACAACAGCATCTCGCAGGCGGTTAATGGCTCGCAGTTCGTGCCG       300
M               TGCCTGCGCCTGAGCCACAACAGCATCTCGCAGGCGGTTAATGGCTCGCAGTTCGTGCCG       300
                ************************************************************

JN377803.1      CTGACCGGCCTGCGAGTGCTTGACCTGTCCTACAACAAGCTGGACCTGTACCATGGGCGC       360
H               CTGACCGGCCTGCGAGTGCTCGACCTGTCCTACAACAAGCTGGACCTGTACCATGGGCGC       360
M               CTGACCGGCCTGCGAGTGCTTGACCTGTCCTACAACAAGCTGGACCTGTACCATGGGCGC       360
                ******************** ***************************************

JN377803.1      TCGTTCACGGAGCTGCCGCAGCTGGAGGCACTGGACCTCAGCTACAACAGCCAG    414
H               TCGTTCACGGAGCTGCCGCAGCTGGAGGCACTGGACCTCAGCTACAACAGCCAG    414
M               TCGTTCACGGAGCTGCCGCAGCTGGAGGCACTGGACCTCAGCTACAACAGCCAG    414
                ******************************************************
```

Figure S7: Representative sequence alignment of the *TLR9* gene (414 bp) between mastitic (M) and healthy (H) ewes compared to reference GenBank accession number (JN377803.1).

```
AF091651.1      GATATCCTTTTCATTGGCAAATGAGGGACCAGGAGAGCCTTTGGGCACTTAGGCCTCTGG       60
H               GATATCCTTTTCATTGGCAAATGAGGGACCAGGAGAGCCTTTGGGCACTTAGGCCTCTGG       60
M               GATATCCTTTTCATTGGCAAATGAGGGACCAGGAGAGCCTTTGGGCACTTAGGCCTCTGG       60
                ************************************************************

AF091651.1      TTCTGTTTTCTGAGAGCTGTATTAGGTCTCAGGAGGGCCCCAGGGGCAGTCTGGGTCAGA       120
H               TTCTGTTTTCTGAGAGCTGTATTAGGTCTCAGGAGGGCCCCAGAGGCAGTCTGGGTCAGA       120
M               TTCTGTTTTCTGGGAGCTGTATTAGGTCTCAGGAGGGCCCCAGGGGCAGTCTGGGTCAGA       120
                ************ ****************************** ****************

AF091651.1      CTCTGGGCAGCATATTGCCTCAGCCAGCTGGACCAGGCTGCCGTGGACCCCGGGCCAGGC       180
H               CTCTGGGCAGCATATTGCCTCTGCCAGCTGGACCAAGCTGCTGTGGACCCCGGGCCAGGC       180
M               CTCTGGGCAGCATATTGCCTCAGCCAGTTGGACCAGGCTGCCGTGGACCCCGGGCCAGGC       180
                ********************* ***** ******* ***** ******************

AF091651.1      AGCGGGCCTTCTTTCAAAACTCCAGGCTGGCTGCTGCGTGCAGACGCAAGGGTCTCCGTC       240
H               AGCGGGCCTTCTTTCAAAACTCCAGGCTGGCTGCTGCGTGCAGACGCAAGGGTCTCCGTC       240
M               AGCGGGTCTTCTTTCAAAACTCCAGGCTGGCTGCTGCGTGCAGACGCAAGGATCTCCGTC       240
                ****** ******************************************** ********

AF091651.1      TGCCTTAACTGGTTCCCAAGCACTTTAGATACCATCTGTACAGTCAAGCTGATCCACAA        299
H               TGCCTCAACTGTTTCCCAAGCACTTTAGATACCATCTGTACAGTCAAGCTGATCCACAA        299
M               TGCCTTAACTGGTTCCCAAGCACTTTAGATACCATCTGTACAGTCAAGCTGATCCACAA        299
                ***** ***** ***********************************************
```

Figure S8: Representative sequence alignment of the *LTF* gene (299 bp) between mastitic (M) and healthy (H) ewes compared to reference GenBank accession number (AF091651.1).

```
FJ901301.1      AAGGGCAAGTCCGAAGAACTTCTGAGAGCTCTGGAAAGCCAAGACTTCCTTCCCACTTCT      60
H               AAGGGCAAGTCCGAAGAACTTCTGAGAGCTCTGGAAAGCCAAGACTTCCTTCCCACTTCT      60
M               AAGGGCAAGTCCGAAGAACTTCTGAGAGCTCTGGAAAGCCAAGACTTCCTTCCCACTTCT      60
                ************************************************************

FJ901301.1      GACTGCGAGGATTTGCTGATGGAATTCATAGAGGTAGATGACAGTGAGGACCAACACCTG      120
H               GACTGCGAGGATTTGCTGATGGAATTCATAGAGGTAGATGACAGTGAGGACCAACACCTG      120
M               GACTGCGAGGATTTGCTGATGGAATTCATAGAGGTAGATGACAGTGAGGACCAACACCTG      120
                ************************************************************

FJ901301.1      ATGCCACACCCCTCCAAAGAACACATGGAGCAAGGCGTGAAGCCCATGCACCTGGATCCT      180
H               ATGCCACACCCCTCCAAAGAACACATGGAGCAAGGCGTGAAGCCCATGCACCTGGATCCT      180
M               ATGCCACACCCCTCCAAAGAACACATGGAGCAAGGCGTGAAGCCCATGCACCTGGATCCT      180
                ************************************************************

FJ901301.1      GACACTGACTCTGGCCGGGGCAGCTGTGACAGCCCTTCGCTCTTGTCTGAAAAGTGTGAT      240
H               GACACTGACTCTGGCCGGGGCAGCTGTGACAGCCCTTCGCTCTTGTCTGAAAAGTGTGAT      240
M               GACACTGACTCTGGCCGGGGCAGCTGTGACAGCCCTTCACTCTTGTCTGAAAAGTGTGAT      240
                ************************************** *********************

FJ901301.1      GAACCTCAGGCCTATCCCTCCAAGTTCCACATTCCAGAGGGCCCTGAGAAGCTGGAGGAT      300
H               GAACCTCAGGCCTATCCCTCCAAGTTCCACATTCCGGAGGGCCCTGAGAAGCTGGAGGAT      300
M               GAACCTCAGGCCTATCCCTCCAAGTTCCACATTCCAGAGGGCCCTGAGAAGCTGGAGGAT      300
                ********************************** *************************

FJ901301.1      CCCGAAACAAATCATACATGTCTCCAGGCCCCTCAGAGCACAAGTGGGGAAGGCAAAATC      360
H               CCCGAAACAAATCATACATGTCTCCAGGCCCCTCAGAGCACAAGTGGGGAAGGCAAAATC      360
M               CCCGAAACAAATCATACATGTCTCCAGGCCCCTCAGAGCACAAGTGGGGAAGGCAAAATC      360
                ************************************************************

FJ901301.1      CCCTATTTTCTGGCCAACGGACCCAAATCTTCCACATGGCCTTTCCCGCAGCCCCCCAGC      420
H               CCCTATTTTCTGGCCAACGGACCCAAATCTTCCACATGGCCTTTCCCGCAGCCCCCCAGC      420
M               CCCTATTTTCTGGCCAACGGACCCAAATCTTCCACATGGCCTTTCCCGCAGCCCCCCAGC      420
                ************************************************************

FJ901301.1      CTGTACAGCCCCAGATATTCTTACCACAACATTGCTGATGTGTGTGAGCTGGCCCTGGGC      480
H               CTGTACAGCCCCAGATATTCTTACCACAACATTGCTGATGTGTGTGAGCTGGCCCTGGGC      480
M               CTGTACAGCCCCAGATATTCTTACCACAACATTGCTGACGTGTGTGAGCTGGCCCTGGGC      480
                ************************************** *********************

FJ901301.1      ATGGCAGGCACCACAGCCACTCTGCTGGACCAAACAGACCAACATGCTTTTAAACCCTCA      540
H               ATGGCAGGCACCACAGCCACTCTGCTGGACCAAACAGACCAACATGCTTTTAAACCCTCA      540
M               ATGGCAGGCACCACAGCCACTCTGCTGGACCAAACAGACCAACATGCTTTTAAACCCTCA      540
                ************************************************************

FJ901301.1      AAAACCATTGAGACTGGCGGGGAAGGAAAGGCAGCCAAACAGAGCGAGTCAGAAGGCTAC      600
H               AAAACCATTGAGACTGGCGGGGAAGGAAAGGCAGCCAAACAGAGCGAGTCAGAAGGCTAC      600
M               AAAACCATTGAGACTGGCGGGGAAGGAAAGGCAGCCAAACAGAGGGAGTCAGAAGGCTAC      600
                ******************************************** ***************

FJ901301.1      AGTTCCGAGCCTGACCAAGACATGGCATGGCCACTGCTCCAAGACAAAACCCCCTTGTTC      660
H               AGTTCCGAGCCTGACCAAGACATGGCATGGCCACTGCTCCAAGACAAAACCCCCTTGTTC      660
M               AGTTCCGAGCCTGACCAAGACATGGCATGGCCACTGCTCCAAGACAAAACCCCCTTGTTC      660
                ************************************************************

FJ901301.1      TCTGCTAAACCCTTGGAATATGTGGAGATCCACAAGGTCAGCCAAGATGGAGTGCTAGCT      720
H               TCTGCTAAACCCTTGGAATATGTGGAGATCCACAAGGTCAGCCAAGATGGAGTGCTAGCT      720
M               TCTGCTAAACCCTTGGAATATGTGGAGATCCACAAGGTCAGCCAAGATGGAGTGCTAGCT      720
                ************************************************************

FJ901301.1      CTGTTCCCAAAACAAAATGAGAAGGTTGACGCCCCTGAAACCAGCAAGGAGTACTCGAAG      780
```

```
H               CTGTTCCCAAAACAAAATGAGAAGGTTGACGCCCCTGAAACCAGCAAGGAGTACTCGAAG       780
M               CTGTTCCCAAAACAAAATGAGAAGGTTGACGCCCCTGAAACCAGCAAGGAGTACTCGAAG       780
                ************************************************************

FJ901301.1      GTGTCTCGGGTGACGGATAGCAACATCCTGGTGTTGATACCGGATCTGCAAGCGCAAAAC       840
H               GTGTCTCGGGTGACGGATAGCAACATCCTGGTGTTGATACCGGATCTGCAAGCGCAAAAC       840
M               GTGTCTCGGGTGACGGATAGCAACATCCTGGTGTTGATACCGGATCTGCAAGCGCAAAAC       840
                ************************************************************

FJ901301.1      CTGACTCTGTTAGAAGAATCAGCCAAGAAGGCCCCGCCAGCCCTGCCATAG       891
H               CTGACTCTGTTAGAAGAATCAGCCAAGAAGGCCCCGCCAGCCCTGCCATAG       891
M               CTGACTCTGTTAGAAGAATCAGCCAAGAAGGCCCCGCCAGCCCTGCCATAG       891
                ***************************************************
```

Figure S9: Representative sequence alignment of the *PRLR* gene (891 bp) between mastitic (M) and healthy (H) ewes compared to reference GenBank accession number (FJ901301.1).

```
XM_060400054.1      CAGCCAGCGACCAGATGAAACATTGGAAGGAGCAGAGGGCCGCGCAGAAACCTGATGTCC   60
H                   CAGCCAGCGACCAGATGAAACATTGGAAGGAGCAGAGGGCCGCGCAGAAACCTGATGTCC   60
M                   CAGCCAGCGACCAGATGAAACACTGGAAGGAGCAGAGGGCCGCGCAGAAACCTGATGTCC   60
                    ********************** *************************************

XM_060400054.1      TGACCACTGGCGCCGGTAATCCAGTAGGAGACAAACTCAATGTTCTGACGGTAGGGCCCC   120
H                   TGACCACTGGCGCCGGTAATCCAGTAGGAGACAAACTCAATGTTCTGACGGTAGGGCCCC   120
M                   TGACCACTGGAGCCGGTAATCCAGTAGGAGACAAACTCAATGTTCTGACGGTAGGGCCCC   120
                    ********** *************************************************

XM_060400054.1      GAGGGCCCCTTCTCGTCCAGGATGTGGTTTTCACTGATGAAATGGCTCACTTTGACCGGG   180
H                   GAGGGCCCCTTCTCGTCCAGGATGTGGTTTTCACTGATGAAATGGCTCACTTTGACCGGG   180
M                   GAGGGCCCCTTCTCGTCCAGGATGTGGTTTTCACTGATGAAATGGCTCACTTTGACCGGG   180
                    ************************************************************

XM_060400054.1      AGAGAATTCCTGAGAGAGTCGTGCACGCCAAAGGAGCAGGGGCTTTTGGCTACTTTGAGG   240
H                   AGAGAATTCCTGAGAGAGTCGTGCATGCCAAAGGAGCAGGGGCTTTTGGCTACTTTGAGG   240
M                   AGAGAATTCCTGAGAGAGTCGTGCACGCCAAAGGAGCAGGGGCTTTTGGCTACTTTGAGG   240
                    ************************* **********************************

XM_060400054.1      TCACACATGACATTACCAGATACTCCAAGGCGAAGGTGTTTGAGCATATTGGAAAGAGGA   300
H                   TCACACATGACATTACCAGATACTCCAAGGCGAAGGTGTTTGAGCATATTGGAAAGAGGA   300
M                   TCACACATGACATTACCAGATACTCCAAGGCGAAGGTGTTTGAGCATATTGGAAAGAGGA   300
                    ************************************************************
```

Figure S10: Representative sequence alignment of the *CAT* gene (300 bp) between mastitic (M) and healthy (H) ewes compared to reference GenBank accession number (XM_060400054.1).

```
JF728302.1      GATGAATGACCTGCAGCGGCGCCTCGGACCCCGGGGCCTGGTCGTACTCGGCTTCCCGTG       60
H               GATGAATGACCTGCAGCGGCGCCTCGGACCCCGGGGCCTGGTGGTACTCGGCTTCCCGTG       60
M               GATGAATGACCTGCAGCGGCGCCTCGGACCCCGGGGCCTGGTCGTACTCGGCTTCCCGTG       60
                ****************************************** *****************

JF728302.1      CAACCAGTTTGGGCATCAGGAAAACGCCAAGAACGAGGAGATCCTGAATTGCCTGAAGTA       120
H               CAACCAGTTTGGGCATCAGGAAAACGCCAAGAACGAGGAGATCCTGAATTGCCTGAAGTA       120
M               CAACCAGTTTGGGCATCAGGAAAATGCCAAGAACGAGGAGATCCTGAATTGCCTGAAGTA       120
                ************************ ***********************************

JF728302.1      CGTCCGACCAGGCGGTGGGTTCGAGCCCAATTTCATGCTCTTCGAAAAGTGCGAGGTGAA       180
H               CGTCCGACCAGGCGGTGGGTTCGAGCCCAATTTCATGCTCTTCGAAAAGTGCGAGGTGAA       180
M               CGTCCGACCAGGCGGTGGGTTCGAGCCCAATTTCATGCTCTTCGAAAAGTGCGAGGTCAA       180
                ********************************************************* **

JF728302.1      TGGCGAGAAGGCGCATCCGCTCTTCGCCTTCCTTCGGGAGA       221
H               TGGCGAGAAGGCGCATCCGCTCTTCGCCTTCCTTCGGGAGA       221
M               TGGCGAGAAGGCGCATCCGCTCTTCGCCTTCCTTCGGGAGA       221
                *****************************************
```

Figure S11: Representative sequence alignment of the *GPX* gene (221 bp) between mastitic (M) and healthy (H) ewes compared to reference GenBank accession number (JF728302.1).

```
XM_060415465.1      CAACAGCGAAAGTCAGGCGGGAGCCGCCCCCGGGCCAGGCCACGGCCCCTCGGGCTTCCC   60
H                   CAACAGCGAAAGTCAGGCGGGAGCCGCCCCCGGGCCAGGCCACGGCCCCTCGGGCTTCCC   60
M                   CAACAGCGAAAGTCAGGCGGGAGCCGCCCCCGGGCCAGGCCACGGCCCCTCGGGCTTCCC   60
                    ************************************************************

XM_060415465.1      GGAAGCTCGGTGCGCGGGTGGCACCAACGTGCCCGGAGCCACCGCCGCGCCCCCTGGCAT   120
H                   GGAAGCTCGGTGCGCGGGTGGCACCAACGTGCCCGGAGCCACCGCCGCGCCCCCTGGCAT   120
M                   GGAAGCTCGGTGCGCGGGTGGCACCAACGTGCCCGGAGCCACCGCCGCGCCCGCTGGCAT   120
                    **************************************************** *******

XM_060415465.1      CCCCACCCCGCGGTGAGAGTATCGGAGGCTACGCAGGCCCCTGAGTGCCAGTGGTGGCGT   180
H                   CCCCACCCCGCGGTGAGAGTATCGGAGGCTACGCAGGCCCCTGAGTGCCAGTGGTGGCGT   180
M                   CCCCACCCCGCGGTGAGAGTATCGGAGGCTACGCAGGCCCCTGAGTGCCAGTGGTGGCGT   180
                    ************************************************************

XM_060415465.1      TGTCCCTTGTCACCTGGAACCCCATGCAGCCGGAACCCAGGCCTAGCGGGGCCGGGGCCC   240
H                   TGTCCCTTGTCACCTGGAACCCCATGCAGCCGGAACCCAGGCCTAGCGGGGCCGGGGCCC   240
M                   TGTCCCTTGTCACCTGGAACCCCATGCAGCCGGAACCCAGGCCTAGCGGGGCCGGGGCCC   240
                    ************************************************************

XM_060415465.1      ATACCCAATTCCTACCCCTGAGATCACAGCGCCCCGAGGGGGCAGGGGACACGGTGATGT   300
H                   ATACCCAATTCCTGCCCCTGAGATCACAGCGCCCTGAGGGGGCAGGGGACACGGTGATGT   300
M                   ATACCCAATTCCTACCCCTGAGATCACAGCGCCCCGAGGGGGCAGGGGACACGGTGATGT   300
                    ************* ******************** *************************

XM_060415465.1      ACGCCTCCACCGAGTGCAAGGCCGAGGTGACGCCCTCCCAGCATGGCAACCGCACCTTCA   360
H                   ACGCCTCCACCGAGTGCAAGGCCGAGGTGACGCCCTCCCAGCATGGCAACCGCACCTTCA   360
M                   ACGCCTCCACCGAGTGCAAGGCCGAGGTGACGCCCTCCCAGCATGGCAACCGCACCTTCA   360
                    ************************************************************
```

Figure S12: Representative sequence alignment of the *Keap1* gene (360 bp) between mastitic (M) and healthy (H) ewes compared to reference GenBank accession number (XM_060415465.1).

```
XM_060402086.1      GGGTTTGGAATACCTGCATAAAAATGGACAGATTCACAGAGATGTGAAAGCTGGCAATAT   60
H                   GGGTTTGGAATACCTGCATAAAAATGGACAGATTCACAGAGATGTGAAAGCTGGCAATAT   60
M                   GGGTTTGGAATACCTGCATAAAAATGGACAGATTCACAGAGATGTGAAAGCTGGCAATAT   60
                    ************************************************************

XM_060402086.1      TCTTCTTGGAGAAGATGGCTCGGTACAGATTGCAGACTTTGGAGTTAGTGCTTTTTTAGC   120
H                   TCTTCTTGGAGAAGATGGCTCGGTACAGATTGCAGACTTTGGAGTTAGTGCTTTTTTAGC   120
M                   TCTTCTTGGAGAAGATGGCTCGGTACAGATTGCAGACTTTGGAGTTAGTGCTTTTTTAGC   120
                    ************************************************************

XM_060402086.1      AACTGGTGGTGACATTACTCGAAATAAAGTGAGAAAAACCTTTGTTGGAACCCCTTGCTG   180
H                   AACTGGTGGTGACATTACTCGAAATAAAGTGAGAAAAACCTTTGTTGGAACCCCTTGCTG   180
M                   AACTGGTGGTGACATTACTCGAAATAAAGTGAGAAAAACCTTTGTTGGAACCCCTTGCTG   180
                    ************************************************************

XM_060402086.1      GATGGCGCCTGAAGTTATGGAACAGGTTCGTGGTTATGACTTCAAAGCTGACATCTGGAG   240
H                   GATGGCGCCTGAAGTTATGGAACAGGTTCGTGGTTATGATTTCAAAGCTGACATCTGGAG   240
M                   GATGGCGCCTGAAGTTATGGAACAGGTTCGTGGTTATGACTTCAAAGCTGACATCTGGAG   240
                    *************************************** ********************

XM_060402086.1      TTTTGGGATCACAGCCATTGAACTGGCCACGGGGGCAGCTCCTTATCATAAGTACCCGCC   300
H                   TTTTGGGATCACAGCCATTGAACTGGCCACGGGGGCAGCTCCTTATCATAAGTACCCGCC   300
M                   TTTTGGGATCACAGCCATTGAACTGGCCACGGGGGCAGCTCCTTATCATAAGTACCCGCC   300
                    ************************************************************

XM_060402086.1      TTTTGGGATCACAGCCATTGAACTGGCCACGGGGGCAGCTCCTTATCATAAGTACCC      357
H                   TTTTGGGATCACAGCCATTGAACTGGCCACGGGGGCAGCTCCTTATCATAAGTACCC      357
M                   TTTTGGGATCACAGCCATTGAACTGGCCACGGGGGCAGCTCCTTATCATAAGTACCC      357
                    *********************************************************
```

Figure S13: Representative sequence alignment of the *OXSR1* gene (357 bp) between mastitic (M) and healthy (H) ewes compared to reference GenBank accession number r (XM_060402086.1).

```
NM_001009429.1      GGAGGCGTAGTCACCGCCGCAGCCACCACCTCCTCCTCAATCATGCCGAAGCACGAGTTC   60
H                   GGAGGCGTAGTCACCGCCGCAGCCACCACCTCCTCCTCAATCATGCCGAAGCACGAGTTC   60
M                   GGAGGCGTAGTCACCGCCGCAGCCACCACCTCCTCCTCAATCATGCCGAAGCACGAGTTC   60
                    ************************************************************

NM_001009429.1      TCCGTGGATATGACCTGTGAAGGCTGCTCTAACGCAGTCACTCGAGTCCTCAACAAGCTA   120
H                   TCCGTGGATATGACCTGTGAAGGCTGCTCTAACGCAGTCACTCGAGTCCTCAACAAGCTA   120
M                   TCCGTGGATATGACCTGTGAAGGCTGCTCTAACGCAGTCACTCGAGTCCTCAACAAGCTA   120
                    ************************************************************

NM_001009429.1      GGAGGAGTTCAATTTGACATTGACCTGCCCAACAAAAAGGTCTGCATCAACTCTGAGCAC   180
H                   GGAGGAGTTCAATTTGACATTGACCTGCCCAACAAAAAGGTCTGCATCAACTCTGAGCAC   180
M                   GGAGGAGTTCAATTTGACATTGACCTGCCCAACAAAAAGGTCTGCATCAACTCTGAGCAC   180
                    ************************************************************

NM_001009429.1      AGCGTGGACACTTTGCTGGAGACCCTGGGGAAAACAGGAAAGGCTGTCTCCTACCTTGGC   240
H                   AGCGTGGACACTTTGCTGGAGACCCTGGGGAAAACAGGAAAGGCTGTCTCCTACCTTGGC   240
M                   AGCGTGGACACTTTGCTGGAGACCCTGGGGAAAACAGGAAAGGCTGTCTCCTACCTTGGC   240
                    ************************************************************

NM_001009429.1      CCCAAGTAGAGAGGCCCCGTCCAGCAGCCTGCAGGATGGACCAGCATGGGCAGGACGCTG   300
H                   CCCAAGTAGAGAGGCCCCGTCCAGCAGCCTGCAGGATGGACCAGCATGGGCAGGACGCTG   300
M                   CCCAAGTAGAGAGGCCCCGTCCAGCAGCCTGCAGGATGGACCAGCATGGGCAGGACGCTG   300
                    ************************************************************

NM_001009429.1      ATCCTCTCCTGCCTTCCAGACAGACCTAGGACCTGGCAATCCCGCTCAGCAATGGTAGTT   360
H                   ATCCTCTCCTGCCTTCCAGACAGACCTAGGACCTGGCAATCCCGCTCAGCAATGGTAGTT   360
M                   ATCCTCTCCTGCCTTCCAGACAGACCTAGGACCTGGCAATCCCGCTCAGCAATGGTAGTT   360
                    ************************************************************

NM_001009429.1      CCTGCGGAGACCGTCACTTGCCCTGCTCCTCTGTAGCTTCCCTGCAATAAAGTCAAGCCG   420
H                   CCTGCGGAGACCCTCACTTGCCCTGCTCCTCTGTAGCTTCCCTGCAATAAAGTCAAGCCG   420
M                   CCTGCGGAGACCGTCACTTGCCCTGCTCCTCTGTAGCTTCCCTGCAATAAAGTCAAGCCG   420
                    ************ ***********************************************

NM_001009429.1      ATTTTGCTGGCGA    433
H                   ATTTTGCTGGCGA    433
M                   ATTTTGCTGGCGA    433
                    *************
```

Figure S14: Representative sequence alignment of the *ATOX1* gene (433 bp) between mastitic (M) and healthy (H) ewes compared to reference GenBank accession number (NM_001009429.1).

```
AJ238319.1      GTGGGCAAGCCCAAGCTGCACTACTTCAACGGACGTGGCAGAATGGAGTGCATTCTTTGG       60
H               GTGGGCAAGCCCAAGCTGCACTACTTCAATGGACGTGGCAGAATGGAGTGCATTCTTTGG       60
M               GTGGGCAAGCCCAAGCTGCACTACTTCAACGGACGTGGCAGAATGGAGTGCATTCGGTGG       60
                **************************** **************************  ***

AJ238319.1      CTCCTGGCTGCAGCCGGAGTGGAGTTTGAAGAGAAATTTATAGAACAACCAGAAGACCTG       120
H               CTCCTGGCTGCAGCCGGAGTGGAGTTTGAAGAGAAATTTATAGAACAACCAGAAGACCTG       120
M               CTCCTGGCTGCAGCCGGAGTGGAGTTTGAAGAGAAATTTATAGAACAACCAGAAGACCTG       120
                ************************************************************

AJ238319.1      GATAAGTTAAAAAATGATGGGAGTTTGATGTTCCAGCAAGTGCCAATGGTTGAAATTGAT       180
H               GATAAGTTAAAAAATGATGGGAGTTTGATGTTCCAGCAAGTGCCAATGGTTGAAATTGAT       180
M               GATAAGTTAAAAAATGATGGGAGTTTGATGTTCCAGCAAGTGCCAATGGTTGAAATTGAT       180
                ************************************************************

AJ238319.1      GGGATAAAGCTGGTGCAGACCAGAGCCATTCTCAACTACGTTGCCACTAAATATAACCTC       240
H               GGGATAAAGCTGGTGCAGACCAGAGCCATTCTCAACTACGTTGCCACTAAATATAACCTC       240
M               GGGATAAAGCTGGTGCAGACCAGAGCCATTCTCAACTACGTTGCCACTAAATATAACCTC       240
                ************************************************************

AJ238319.1      TACGGGAAAGACATGAAGGAGAGAGCCCTGATTGATATGTACTCAGAGGGTGTGGCAGAT       300
H               TACGGGAAAGACATGAAGGAGAGAGCCCTGATTGATATGTACTCAGAGGGTGTGGCAGAT       300
M               TACGGGAAAGACATGAAGGAGAGAGCCCTGATTGATATGTACTCAGAGGGTGTGGCAGAT       300
                ************************************************************

AJ238319.1      TTGGGTGAAATGATCCTGCTTTTGCCACTGTGCCCACCTGATCAAAAAGATGCCAAAATA       360
H               TTGGGTGAAATGATCCTGCTTTTGCCACTGTGCCCACCTGATCAAAAAGATGCCAAAATA       360
M               TTGGGTGAAATGATCCTGCTTTTGCCACTGTGCCCACCTGATCAAAAAGATGCCAAAATA       360
                ************************************************************

AJ238319.1      GCTCAGATCAAACAGAGTACAACAAACCGTTATCTCCCTGCATTTGAAAAAGTGCTGAAG       420
H               GCTCAGATCAAACAGAGTACAACAAACCGTTATCTCCCTGCATTTGAAAAAGTGCTGAAG       420
M               GCTCAGATCAAACAGAGTACAACAAACCGTTATCTCCCTGCATTTGAAAAAGTGCTGAAG       420
                ************************************************************

AJ238319.1      AGCCACGGACAAGACTATCTTGTGGGCAACAAGCTGAGCAAGGCTGACATCCACCTGGTT       480
H               AGCCACGGACAAGACTATCTTGTGGGCAACAAGCTGAGCAAGGCTGACATCCACCTGGTT       480
M               AGCCACGGACAAGACTATCTTGTGGGCAACAAGCTGAGCAAGGCTGACATCCACCTGGTT       480
                ************************************************************
```

Figure S15: Representative sequence alignment of the *GST* gene (480 bp) between mastitic (M) and healthy (H) ewes compared to reference GenBank accession number (AJ238319.1).

```
XM_012132935.5      CACTGAACACAACAAGTCCAAGCATGGCATCACCAGACCACTCAGTGGAATCTTCTATCT   60
H                   CACTGAACACAACAAGTCCAAGCATGGCATCACCAGACCACTCAGTGGAATCTTCTATCT   60
M                   CACTGAACACAACAAGTCCAAGCATGGCATCACCAGACCACTCAGTGGAATCTTCTATCT   60
                    ************************************************************

XM_012132935.5      ATGGAGACACATTGCTTGGCTTCAGTGATTCTGAAATGGAAGAGATAGATAGTGCCCCTG   120
H                   ATGGAGACACATTGCTTGGCTTCAGTGATTCTGAAATGGAAGAGATAGATAGTGCCCCTG   120
M                   ATGGAGACACATTGCTTGGCTTCAGTGATTCTGAAATGGAAGAGATAGATAGTGCCCCTG   120
                    ************************************************************

XM_012132935.5      GAAATGTCAAACAGAAGGGTCCCAAAACACAGTCAGTGTGGCCTTCTGGGGACCCAGTCC   180
H                   GAAATGTCAAACAGAAGGGTCCCAAAACACAGTCAGTGTGGCCTTCTGGGGACCCAGTCC   180
M                   GAAATGTCAAACAGAAGGGTCCCAAAACACAGTCAGTGTGGCCTTCTGGGGACCCAGTCC   180
                    ************************************************************

XM_012132935.5      AACCTTTGTCATCATCACAAGGGAACAGCGCTGCAGCACATGATTCCCAGTGTGAAAATG   240
H                   AACCTTTGTCATCATCACAAGGGAACAGCGCTGCAGCACATGATTCCCAGTGTGAAAATG   240
M                   AACCTTTGTCGTCATCACAAGGGAACAGCGCTGCAGCACATGATTCCCAGTGTGAAAATG   240
                    ********** *************************************************

XM_012132935.5      CACCAAAGAAAGAAGTGCCTGTAAGTCCTGGTCATCGAAAAACCCCATTCACAAAAGACA   300
H                   CACCAAAGAAAGAAGTGCCTGTAAGTCCTGGTCATCGAAAAACCCCATTCACAAAAGACA   300
M                   CACCAAAGAAAGAAGTGCCTGTAAGTCCTGGTCATCGAAAAACCCCATTCACAAAAGACA   300
                    ************************************************************

XM_012132935.5      AACATACAAGCCGCTTGGAGGCTCACCTCACAAGAGATGA     340
H                   AACATACAAGCCGCTTGGAGGCTCACCTCACAAGAGATGA     340
M                   AACATACAAGCCGCTTGGAGGCTCACCTCACAAGAGATGA     340
                    ****************************************
```

Figure S16: Representative sequence alignment of the *Nrf2* gene (340 bp) between mastitic (M) and healthy (H) ewes compared to reference GenBank accession number (XM_012132935.5).
